# Salinity tolerance mechanisms of an Arctic Pelagophyte using comparative transcriptomic and gene expression analysis

**DOI:** 10.1038/s42003-022-03461-2

**Published:** 2022-05-25

**Authors:** Nastasia J. Freyria, Alan Kuo, Mansi Chovatia, Jenifer Johnson, Anna Lipzen, Kerrie W. Barry, Igor V. Grigoriev, Connie Lovejoy

**Affiliations:** 1 Département de biologie, Institut de Biologie Intégrative et des Systèmes, Université Laval, Québec, Canada; 2 Québec Océan, Département de biologie, Université Laval, Québec, Canada; 3 U.S. Department of Energy Joint Genome Institute, Lawrence Berkeley National Laboratory, Berkeley, CA 94720 USA; 4 Department of Plant and Microbial Biology, University of California Berkeley, Berkeley, CA 94720 USA

**Keywords:** Gene expression, Transcriptomics, Salt

## Abstract

Little is known at the transcriptional level about microbial eukaryotic adaptations to short-term salinity change. Arctic microalgae are exposed to low salinity due to sea-ice melt and higher salinity with brine channel formation during freeze-up. Here, we investigate the transcriptional response of an ice-associated microalgae over salinities from 45 to 8. Our results show a bracketed response of differential gene expression when the cultures were exposed to progressively decreasing salinity. Key genes associated with salinity changes were involved in specific metabolic pathways, transcription factors and regulators, protein kinases, carbohydrate active enzymes, and inorganic ion transporters. The pelagophyte seemed to use a strategy involving overexpression of Na^+^-H^+^ antiporters and Na^+^ -Pi symporters as salinity decreases, but the K^+^ channel complex at higher salinities. Specific adaptation to cold saline arctic conditions was seen with differential expression of several antifreeze proteins, an ice-binding protein and an acyl-esterase involved in cold adaptation.

## Abstract (web summary)

Arctic microalgae show specific adaptation to cold saline arctic conditions with differential expression of several antifreeze proteins, an ice-binding protein and an acyl-esterase involved in cold adaptation.

## Introduction

Unicellular eukaryotic microalgae are ubiquitous in the global ocean and have a central role in photosynthesis, carbon cycling, and marine biogeochemical cycles. Oceans and coastal regions are experiencing an increase in freshening events linked to recent climate change^1^, but the impact of freshening on marine species is little studied. Research on salinity tolerance in microalgae has focused on a few freshwater species that are halotolerant, and when stressed under saline conditions are of interest for biofuel and bioenergy production^2,3^. In contrast, marine microalgae are mostly excluded from freshwaters. The few marine species that have invaded freshwaters over evolutionary time scales, are mostly related to Arctic species^4^. Arctic algae may be predisposed for such invasions given that sea-ice melting and freezing results rapid and marked changes in salinity, making Arctic species good models to examine genomic adaptation to variable salinity.

In the Arctic, high salinities are found in brine channels that form in sea-ice during freezing and persist throughout winter^5^. In contrast, low salinities occur when sea-ice melts during spring and summer, with surface salinities sometimes approaching freshwater levels^6^. To survive in fluctuating saline environments, microorganisms need to modify cellular, physiological, and metabolic processes^7^, but few studies have directly addressed the genomic basis or mechanisms for tolerance to either higher or lower salinities that are found in the sea-ice-associated environment^8^. Arctic marine ecosystems are characterized not only by strong seasonality but also interannual variability of environmental factors, especially sea-ice cover^9^. With global warming, the Arctic Ocean is overall becoming less salty due to melting permafrost contributing to increased river flow, and loss of multiyear ice^10^. This freshening contributes to increasing variability in the timing and character of sea-ice formation and melt, exposing photosynthetic microbial communities to increased salinity fluctuations over shorter periods^11^. This is of concern as salt stress and osmotic shock are among the main factors limiting the growth and productivity of plants and microorganisms^12^. Overall, there is a pressing need to investigate salinity tolerances in Arctic species given the rapid changes now occurring in the Arctic^1^.

We focus our study on a specific marine microalga from the western side of Northern Baffin Bay (Arctic, Canada). The pelagophyte (CCMP2097) originated from a sample of liquid water from a ca. 10-cm^3^ pocket just under the upper surface of consolidated sea‑ice. The water in the pocket was assumed to be saline because of brine release, but salinity was not measured at that time. The alga has been maintained at 4 °C in an L1 seawater medium (salinity 33–35) since collection. All known pelagophytes are marine^13,14^ with benthic coastal and picoplanktonic species that, with some exceptions, are in the Orders of Sacinochysidales and Pelagomonadales, respectively. Most described species of pelagophytes are in the Sarcinochysidales, which are found in diverse habitats including marine sands and as epibionts on marine organisms^14^ and can be capsoid or filamentous. In contrast smaller globally distributed unicellular picoplankton species are mostly in the Pelagomonadales and found in the open ocean^15^ but are rarer in the Arctic^16^. Several single-celled pelagophytes are common in the water column of coastal regions^17^, and some species, such as *Aureococcus anophagefrens*, (Pelagomonadales) and *Aureoumbra* (Arcinochysidales or Sacinochysidales) are responsible for brown tides in the northeast Atlantic Ocean and elsewhere^18^. Although pelagophytes have been occasionally reported from the Arctic^19–21^, no harmful algal events have been attributed to pelagophytes to date. Among the Arctic pelagophytes are several taxonomically undescribed clades including one at the base of Pelagomonadales that includes CCMP2097, and to date has only been found in the Arctic^22,23^.

The aim of this study was to determine the range and response of CCMP2097 to changes in salinity, to gain a deeper understanding of the potential genetic capacity of an ice-associated Arctic alga to adjust to a range of realistic salinities that would be encountered in its ice influenced habitat. For this, we grew the isolate at salinities from 45 to 8 and used RNA-Seq transcriptomics to describe differential expression profiles. Based on research in plants, we hypothesized and found that transcription factors and regulators, protein kinases, carbohydrate-active enzymes, transporters, secondary metabolites, and secretion were involved in the acclimation of the pelagophyte to a range of high to low salinities. Specifically, we show overexpression of Na^+^–H^+^ antiporters and Na^+^–Pi symporters as salinity decreases, but with K^+^-channel complex overexpressed at higher salinities. More surprisingly, we found the potential implications of ice-binding proteins and antifreeze proteins with differential expression under the tested salinity conditions, suggesting tight coupling between cold temperature adaptation and salinity. We speculate that these key genes confer properties to adapt to progressive changes in salinity and facilitate survival in the sea-ice-influenced ocean.

## Results

All results are from the pelagophyte culture CCMP2097 grown at ca. 4 °C and 100 µmol photons m^−2^ s^−1^. Starting from salinity (S.) 45 (t1) then, progressively replacing the culture medium after each sub-sampling: going from 35 (t2); to 25 (t3); to 16 (t4); and to 8 (t5) (Supplementary Fig. 1). In parallel, control cultures followed the same media replacement strategy, renewing the S.45 media along the same time frame (tc1 to tc5). All treatments were carried out in triplicate using three separate flasks per treatment, which were individually tracked over the experiment.

### Cell vitality

The proportion of live cells compared to dead varied from 61 to 97%, with two exceptions: one replicate at tc1–S.45 and one at t5–S.8 were <30% live cells (Supplementary Table 1 and Supplementary Fig. 2a). Growth rates over the experiment were less than 0.3 d^–1^ but with slightly lowered growth rates when first exposed to t3–S.25 and t5–S.8. Cellular Chl *a* concentration under the different salinities varied, fluctuating around 0.02 pg chl cell^–1^. For the constant S.45 controls, where due to logistic constraints only one of the triplicates was analyzed for each transfer, the Chl *a* per cell tended to be higher (0.04–0.09) except for the last transfer where concentrations fell to 0.02 (Supplementary Table 1 and Supplementary Fig. 2b).

Nutrients remained replete throughout the experiment and were never below 179 µmol L^−1^ for Nitrate. Phosphate was drawn down over the course of the experiment, especially in the last two lower salinity treatments with the lowest value of 0.24 µmol L^−1^ (Supplementary Table 2 and Supplementary Fig. 2c, d). Nutrient consumption varied slightly and was significant (repeated measures ANOVA, *F* = 19.3 *P* value <0.05) between time 4 and time 5, but with no significant trend over the experiment (Supplementary Table 3).

A Constrained Correspondence Analysis (CCA) based on ancillary pigment to Chl *a* ratios separated cells grown under lower salinities (8, 16, and 25) from the higher salinities (35 and 45), which were more dispersed (Supplementary Table 4 and Supplementary Fig. 3). The Chl *a* degradation pigment phaeophytin and photoprotective pigments, diadinoxanthin and zeaxanthin, separated along the first axis which was significant (ANOVA, *F* = 2.4, *P* value <0.01). Total explanatory value was 30% of the variance, with significant explanatory variables being salinity at the time of sampling (*R*^2^ = 0.8, *P* value <0.001) and the concentration of Chl *a* per cell (*R*^2^ = 0.5, *P* value <0.01).

### An overview of transcriptome assembly and annotation

**Fig. 1:**
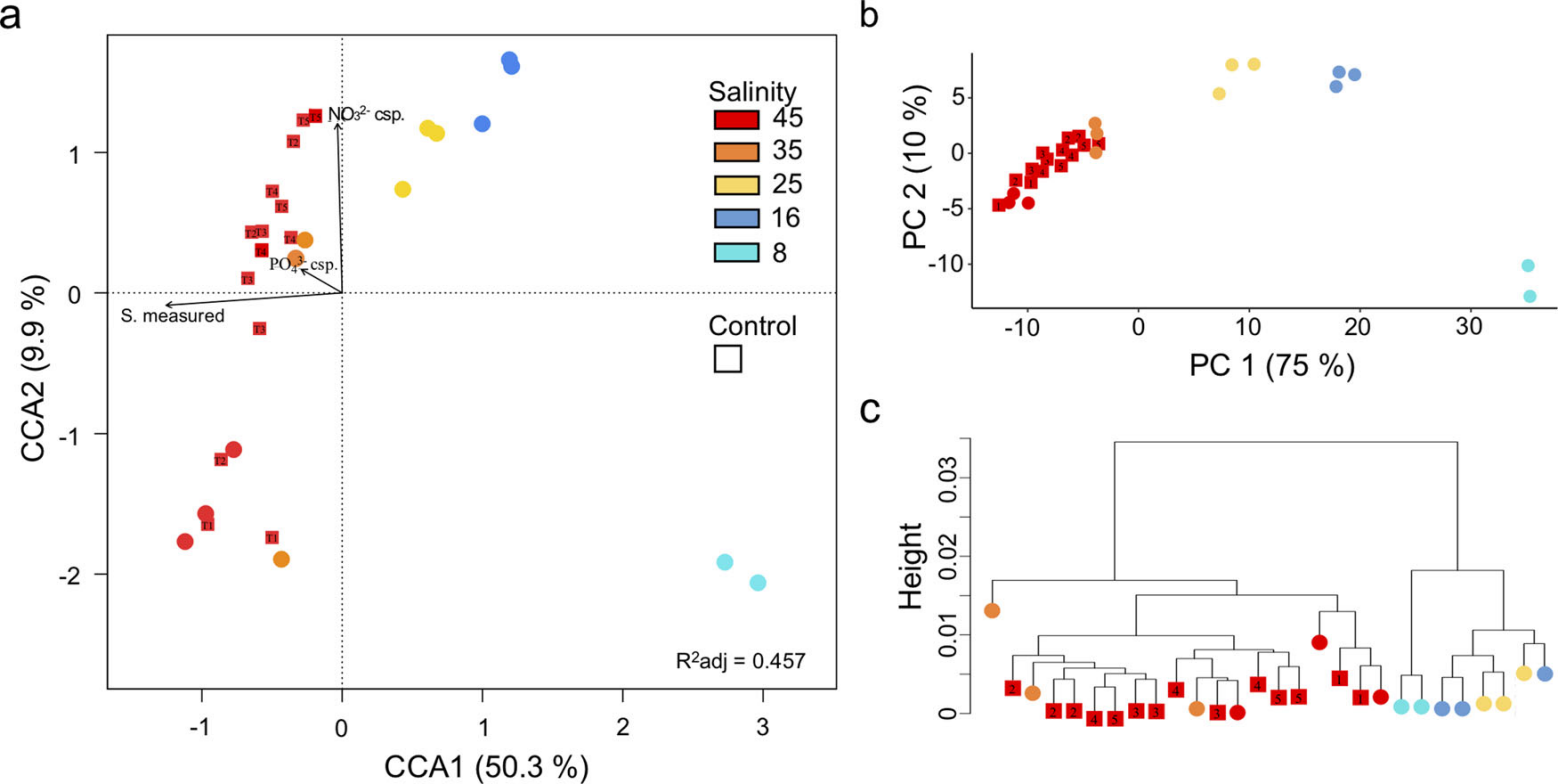
Clustering of transcriptome samples with normalized reads counts. Experimental salinity values are indicated by colored circles, with S.45 controls as red squares. The number in each square represents the time of sampling (tc1 to tc5). **a** CCA with the Bray-Curtis dissimilarity measure of reads per gene of all samples. Arrows indicate significant correlations between culture conditions and samples of each salinity (S). **b** Transcriptome reads per gene transformed to a differential gene expression matrix and clustered using Principal Component Analysis (PCA) and **c** dendrogram was constructed using the complete linkage method from *stats* package on R.

**Fig. 2:**
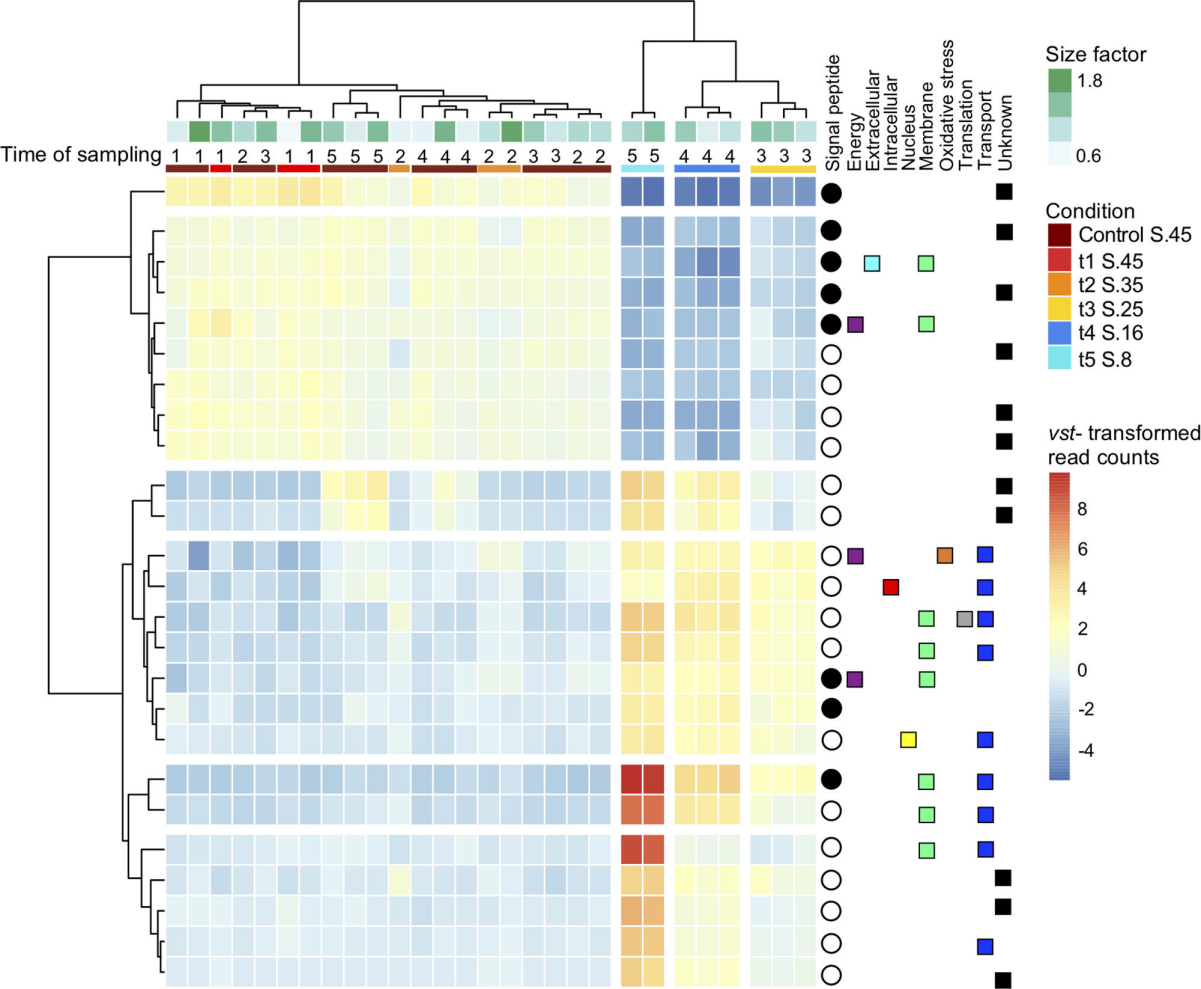
Top 25 genes with the most variant expression. Heatmap of clustered results based on normalized and transformed read counts with *vst()* function in *DESeq2* package on R. Size factor (greens) correspond to the sequencing depths of each transcriptome. Condition (bars beneath time of sampling) corresponds to salinity treatment, control condition is S.45, the number above is the sampling time. The annotation of each gene is on the right, indicated by squares, with each category having a separate color. Unknown function genes are in black. Genes with signal peptides shown with filled circle, the empty circle indicates no signal peptide was found. Note that the lowest salinity (S.8, aqua bar) is arranged between the higher and mid salinities.

Overall, the transcriptomes generated from ca. 9 million to 47 million clean reads for each sample (Supplementary Table 5). All 28 transcriptomes were mapped against the CCMP2097 genome^24^, which contains 19,402 annotated gene models. The same treatment clustering pattern emerged using three methods: (1) based on a normalized standardized CCA (Fig. 1a); (2) using transformed count data to the log_2_ scale for principal component analysis (PCA; Fig. 1b); and (3) where a dendrogram was constructed using the complete linkage method (Fig. 1c). The three methods all indicated that the higher salinities (S.35 and S.45) were dispersed, but clearly separated from lower salinity treatments (S.25, S.16, and S.8), which was statistically significant (ANOVA: *F* = 13.2, *P* value <0.001). Among the culture condition variables, the salinity measured at the time of sampling (*R*^2^ = 1, *P* value <0.001) and total nitrate consumption (*R*^2^ = 0.9, *P* value <0.001) were significant.

### Differentially expressed genes analysis

To identify differentially expressed genes (DEGs), we used two methods of analysis: the DOE JGI pipeline and our customized pipeline (see “Methods”). The two methods yielded broadly similar trends when comparing salinity change treatments (Supplementary Table 6). However, we found more overexpressed genes using our customized pipeline and the remaining analysis will focus on these broader results. To identify the DEG from the entire dataset, the criteria for a fold change was >2 (|log_2_ | FC > 2) and significant DEGs were selected using the adjusted *P* value of <0.05. Our initial inspection of the top 25 most variant DEGs showed that eleven were of unknown function with eight of these including a signal peptide, which is a short sequence of amino acids used to target the protein towards the secretory pathway^25^ (Fig. 2). Eight of the top 25 DEGs were assigned to membrane functions and another nine were annotated as transporters. Several genes were annotated for multiple functions with five genes as both membrane and transport, and one was also annotated as involved in translation. Three genes were involved in energy production and conversion. The top 25 DEG clusters revealed a clear pattern of expression of specific genes associated with high (45, 35), medium (25, 16), and low (8) salinities (Fig. 2).

The gene expression of the control S.45 samples changed little over time, except for tc1 compared to tc2 (Supplementary Fig. 4a). The initial difference in DEGs may have been related to the poor condition of the tc1 cultures (Supplementary Fig. 1a and Supplementary Table 1), which had been most recently moved between incubators. In addition, only two triplicates of the tc1 were successfully sequenced (Supplementary Table 5). Following this initial perturbation, there were few DEGs in the S.45 controls over the remaining experiment (Supplementary Fig. 4b).

**Fig. 3:**
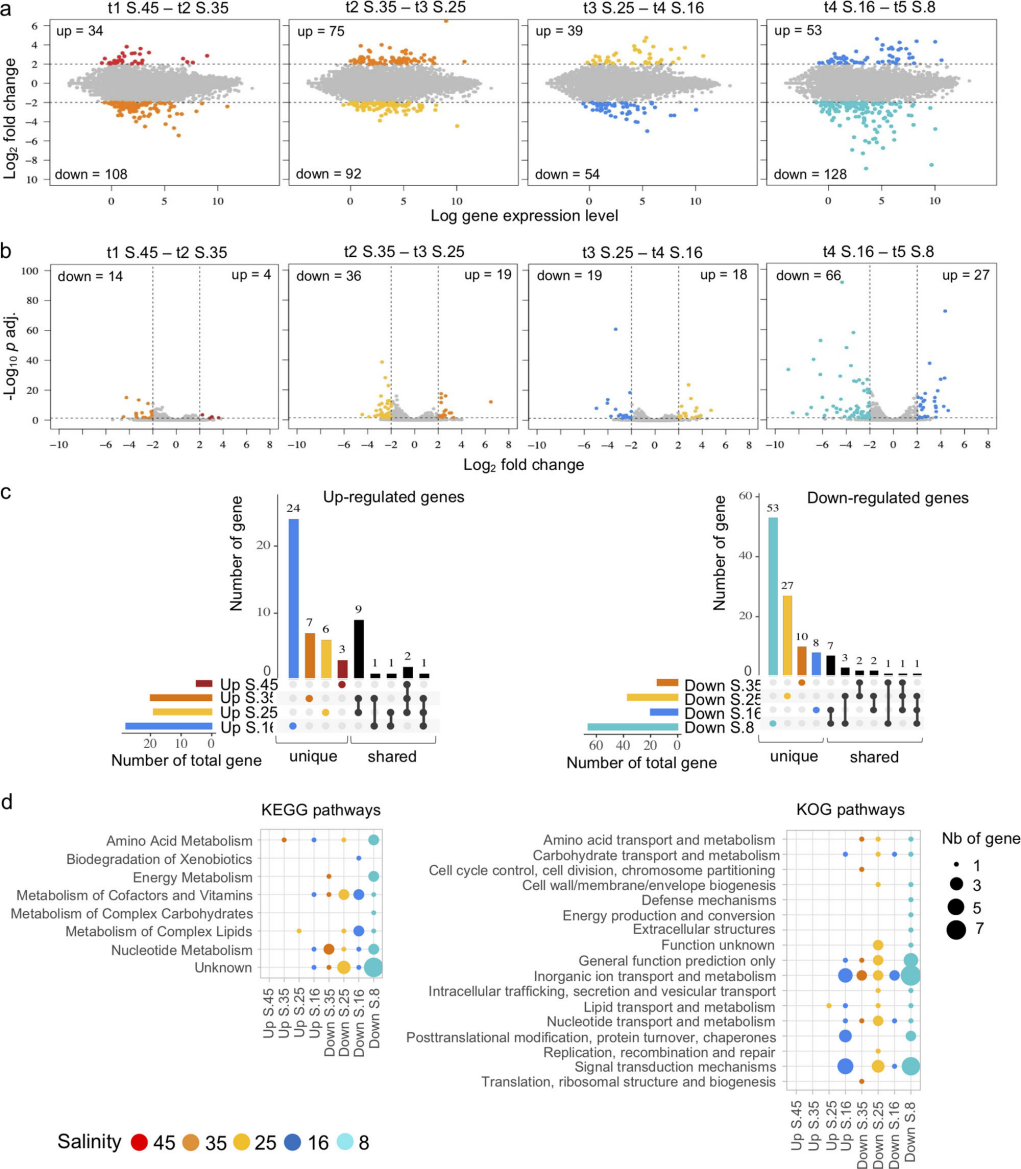
Differential expression genes from a progressive change of salinity. **a** Comparison scatter plot of RNA-Seq analyses of the progressive salinity change. Each point represents a unigene. Points higher than 2 of log_2_ fold change indicate genes upregulated and each point lower than −2 of log_2_ fold change indicate genes downregulated. Each number of up and downregulated genes are indicated. **b** Scatter plot of filtered up- and downregulated genes with an adjusted *P* value of <0.05. **c** UpsetR plot indicated shared and unique genes between the comparison of the condition of salinity. **d** KEGG and KOG pathways of up- and downregulated genes of each comparison between the change from a higher to lower salinity. Salinity treatments are indicated by colors from highest salinity (S.45) in red to lowest salinity (S.8) in turquoise.

DEGs were detected between all salinity changes (Fig. 3a and Supplementary Table 6). By convention points with +2 of log2 fold change or higher are referred to as upregulated and those with –2 of log2 fold or higher are referred to as downregulated for the specific comparison^26^. Significant upregulated DEGs went from a minimum of 4 at t1–S.45 compared to t2–S.35 and maximum of 27 at t4–S.16 compared to t5–S.8 (Fig. 3b). The MA plots (Fig. 3a) show mean expression compared with log-fold changes for the selected comparison and the and volcano plots (Fig. 3b) compares log-fold changes with adjusted *p* values. The fewest downregulated significant DEGs (14) were detected in t2–S.35 (compared to t1–S.45) and the maximum (66) were detected in t5–S.8 compared to t4–S.16 (Fig. 3b). Following total DEGs, there were more significant downregulated DEGs overall. We found more unique than shared DEGs within all salinity comparisons (Fig. 3c). There were 25 total DEGs of opposite sense (up or down) shared and most were of unknown function (Supplementary Table 7).

### Weighted gene co-expression network analysis (WGCNA)

We first explored the expression patterns of 3969 significant DEGs detected in all controls (*n* = 14) using WGCNA. The analysis placed these into 21 modules according to the similarity of expression patterns of each gene in the hierarchical clustering dendrogram (Supplementary Fig. 5a). None of the 21 modules were significantly correlated with a temporal sampling effect (Supplementary Fig. 5b). The module network dendrogram of eigengenes distance, where the eigengenes represent the gene expression profile of the entire module (Supplementary Fig. 5c), showed two main branches where seven related modules separated tc1 from the other controls.

In a second WGCNA, we analyzed the expression patterns of 3977 significant DEGs among all samples (*n* = 28), which formed 12 modules (Supplementary Fig. 6a). In the Pearson’s rank correlation between modules, salinity change had the greatest impact on DEGs (Supplementary Fig. 6b). The time effect was driven by the salinity change and the module network dendrogram (Supplementary Fig. 6c) shows two main branches correlated with higher and lower salinities. Further exploration of the WGCNA results to identify gene clusters associated with the modules was precluded by the low number of samples.

### Functional annotations

To further glean biological meaning from the DEGs resulting from the salinity changes, we examined the placement of genes within KEGG, KOG, and GO databases. There were no upregulated DEGs in KEGG or KOG-annotated pathways detected at S.45 compared to S.35 (Fig. 3d and Supplementary Table 8). Overall, there were fewer KEGG pathway genes upregulated compared to downregulated (9 versus 69, Supplementary Table 8). The greatest numbers of DEGs were placed “Unknown KEGG pathway” these were mostly downregulated DEGs at S.25 and S.8. Annotated KEGG pathway genes were also mostly downregulated (Fig. 3c and Supplementary Table 8) with DEGs found in all pathways for S.8, except “Biodegradation of Xenobiotics”.

In KOG pathways, upregulated DEGs were mostly found for the S.16 condition (S.16 to S.8 comparison). All pathways, except for three, included downregulated DEGs at S.8. There were 11 of the 17 pathways downregulated at S.25 compared to S.35. Notably “Inorganic ion transport and metabolism” was consistently downregulated over all comparisons, with maximum DEGs reads at S.8 (Fig. 3d). The GO terms also showed the most upregulated DEGs in S.16 and the most downregulated at S.8 (Supplementary Figs. 7 and 8). Within the category of “Cellular Components”, membrane-associated genes were highly sensitive with upregulated expression at the higher salinities and more DEGs downregulated over the dilution series with a maximum at S.8 (Supplementary Fig. 7). In the GO “Biological Process” and “Molecular Function” categories, the greatest number of upregulated genes were seen at S.16. For downregulated genes different process were implicated at the different points, for example at S.25 there were multiple DEGs associated with carbohydrate, metabolic, and nucleotide catabolic processes in the “Biological Process” terms and hydrolases in the “Molecular Function” terms. Among others, significant DEGs at S.8 in ‘Biological Process’ were involved with transport generally, and chloride, cation, electron, and proton transports, whereas ion transport was upregulated at S.16 (Supplementary Fig. 7). In the “Molecular Function” category, which indicates enzyme activity, we found DEGs in “voltage-gated chloride” downregulated at S.8 and “voltage-gated potassium channel activity” upregulated at S.16 (Supplementary Fig. 8).

### DEGs between the highest and the lowest salinity

**Fig. 4:**
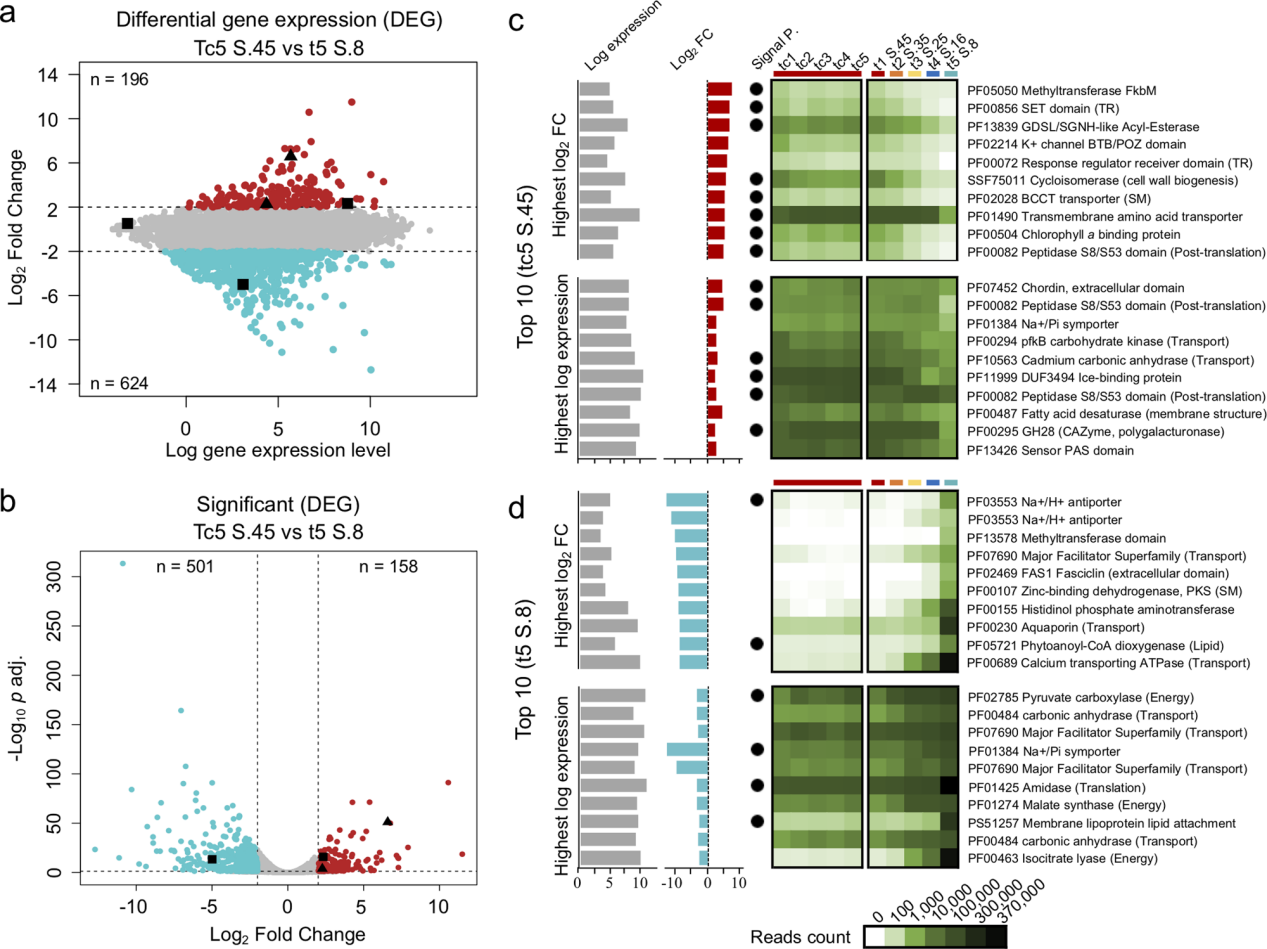
Differential expression genes (DEGs) at the highest and the lowest salinities. Black squares indicate ice-binding protein and black triangles indicate protein involved in freezing/cold acclimation. **a** Comparison scatter plot of RNA-Seq analyses of tc5–S.45 (red points) and t5–S.8 (blue points). Each number (*n*) of overregulated differentially expressed genes are shown. **b** Scatter plot of significant DEGs with an adjusted *P* value of <0.05. **c**, **d** Read counts of the top 10 log2 fold change and highest log expression genes under the two extreme conditions, the read counts under all conditions are indicated in the heatmap. **c** Barplots indicate the top ten of tc5–S.45 significant DEGs ordered by the highest log_2_ fold change (log_2_ FC) and the highest log gene expression level. **d** Lower panel indicate the top ten of up- and downregulated genes ordered by the log gene expression level. The presence of a signal peptide (P.) is indicated with a black circle.

To identify changes more clearly at the two extreme conditions (S.45 and S.8) we compared DEGs from the same sampling time (tc5 and t5; Fig. 4a). Overall, 158 DEGs (at tc5– S.45) and 501 DEGs (at t5–S.8) were significant (Fig. 4b). Among these were three genes coding for an ice-binding protein (IBP, PF11999) with one significant DEG at S.8 and one significant DEG at S.45. Two genes annotated as an acyl-esterase (PF13839) involved in freezing resistance/cold acclimation^27^ were significantly overexpressed at S.45.

Selecting the greatest fold change DEGs (the top 10 genes ordered by log_2_ FC at tc5–S.45 compared to t5–S.8, two DEGs were predicted to be transcription regulators (TRs) of different domains (Fig. 4c) and included a conserved SET domain (PF00856) and a response regulator membrane receiver domain (PF00072). In the top "ten genes, ordered by higher to lower expression level at tc5–S.45, we retrieved one DEG predicted to be an IBP, and one DEG annotated as sodium (Na^+^) –inorganic phosphate (Pi) symporter (PF01384).

At t5–S.8, the top ten genes with greater than log_2_ FC ordered by rank were mostly genes involved with Na^+^ and potassium (K^+^; PF03553) transport, other major facilitator transporters (PF07690) and an aquaporin (water channel; PF00230). In the top ten genes ordered by expression level at S.8, three out of the ten DEGs were involved in energy metabolism (PF02785, PF01274, and PF00463), two were major facilitator transporters (PF07690) and one involved in membrane lipid attachment (PROSITE database: PS51257).

We then compared tc5–S.45 versus t5–S.8 DEGs placement in KEGG, KOG, and GO databases. Among the KEGG and KOG classes, more DEGs were involved in carbohydrate metabolism, lipid, secondary metabolites, energy, transporter, and defense mechanisms at the lowest salinity (Supplementary Figs. 9 and 10). Similarly, for GO term enrichments, we retrieved more DEGs at the lowest salinity. Among these were DEGs involved with stress responses, such as pH regulation (Supplementary Figs. 11–16).

### DEGs of specific pathways

**Fig. 5:**
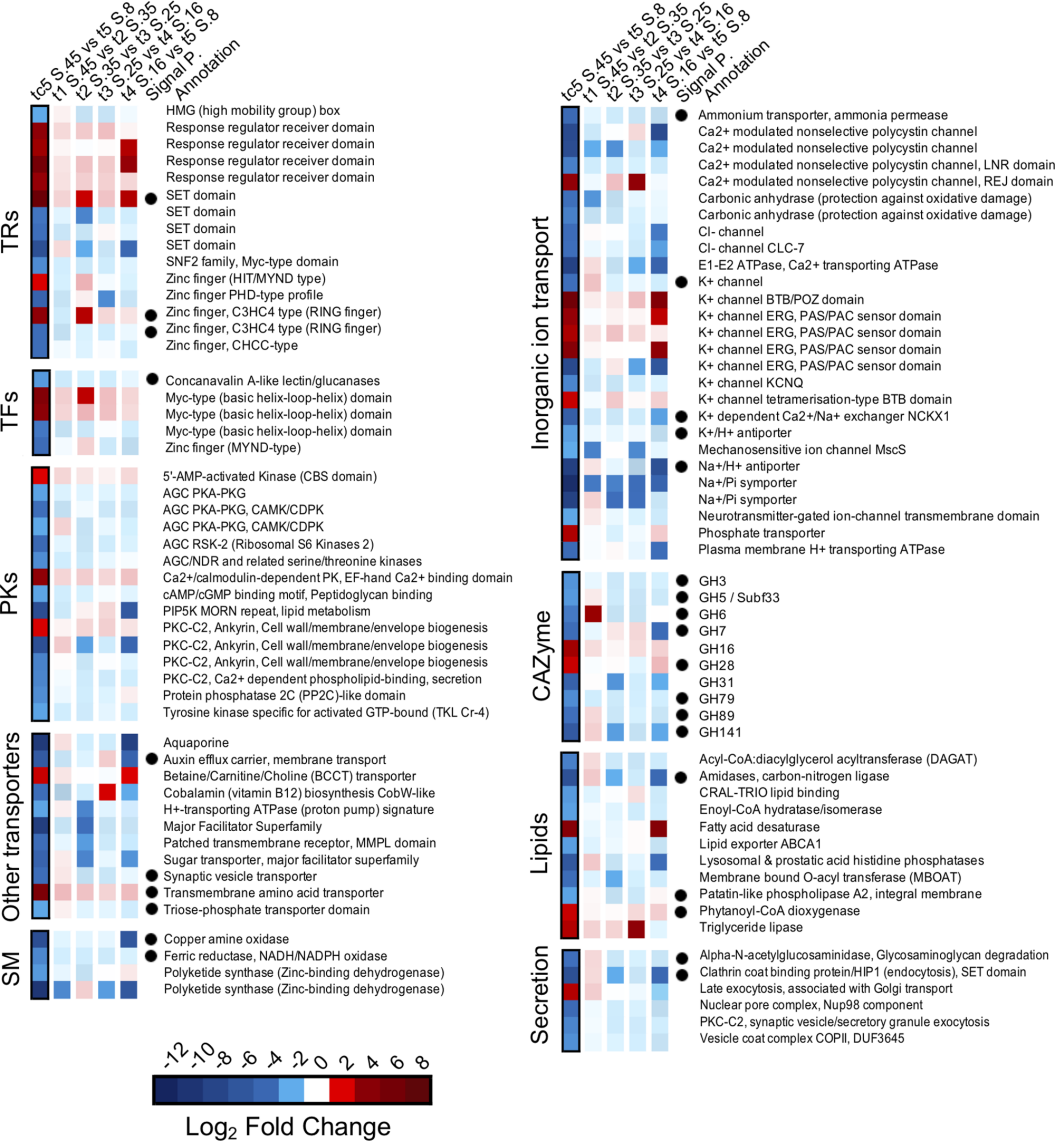
Differential expression of genes involved in nine selected PFAM superfamily or PROSITE categories. The log_2_ fold change for each comparison of salinity (see Figs. 3 and 4) are indicated by the blue and red colors. The intensity of overexpression between the salinity comparisons follows the convention that reds indicate greater expression at the higher salinity, and blues indicate greater expression at the lower salinity of the comparison. The first column compares S.45 to S.8. and red indicates overexpression at S.45 and blue indicates overexpression at S.8. The presence of a signal peptide (P.) is indicated with a black circle. TRs transcription regulators, TFs transcription factors, PKs protein kinases, SM secondary metabolites, CAZYmes carbohydrate-active enzymes. CAZYmes glycoside hydrolase (GHs) details are given in Supplementary Table 9.

**Fig. 6:**
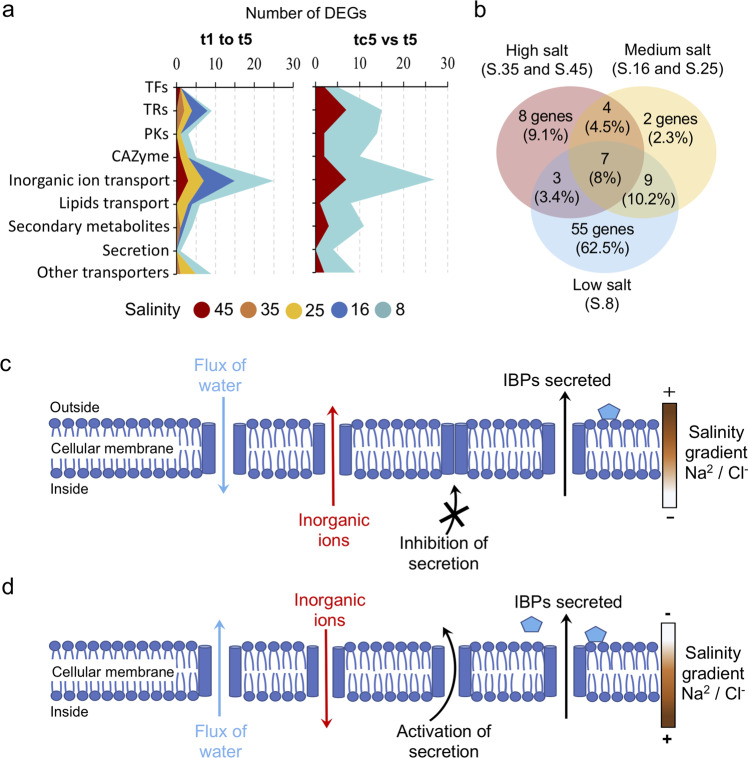
Comparison of the nine selected pathways and potential actions of this pelagophyte strategy to tolerate salinity fluctuations. **a** Number of differentially expressed genes (DEGs) overexpressed at each time of sampling (t1 to t5) for progressive decreased salinity change and between tc5–S.45 (control) and t5–S.8 among the different categories. **b** Venn diagram shows how many functions from all categories mentioned above that were shared and unique between high (group of S.35 and S.45), medium (S.16 and S.25) and low salinity (S.8). The potential strategy of pelagophyte when exposed at (**c**) high and (**d**) low salt. Blue pentagon symbol represents ice-binding protein (IBP). TFs transcription factors, TRs transcription regulators, PKs protein kinases, CAZYmes carbohydrate-active enzymes.

Transcription factors (TFs), transcription regulators (TRs), protein kinase (PKs), carbohydrate-active enzymes (CAZymes), transporters of inorganic ions, lipids, secondary metabolites, and secretion play crucial roles in signal transduction pathways involved in essential biological processes. There are 687 TR-TF genes annotated in the CCMP2097 genome. Among those, we found DEGs annotated as being in the response regulator membrane receiver domain (PF00072) with the greatest log-fold changes at S.45 compared to S.8 and three out of four overexpressed SET domain DEGs (PF00856) for S.8 (Fig. 5); the overexpressed DEG at S.45 included a signal P. In total, 15 TRs and 5 TFs were significantly differentially expressed among all salinities (Figs. 5 and 6). For PKs, we identified 15 DEGs in total, mostly overexpressed at S.8 (Figs. 5 and 6). Some of the major PKs in our dataset included one PKC-C2 (Ca^2+^-dependent phospholipid-binding protein, PF00168) involved in secretion (KOG1028) and three PKC-CK2 identified as an ankyrin protein involved in cell-wall biogenesis (KOG4177).

The CCMP2097 genome has in total 260 genes predicted to be CAZymes, ten of these were classed as DEGs (Figs. 5 and 6), with most being glycoside hydrolases (GHs; Supplementary Table 9). Two genes (GH16 and GH28) were overexpressed at S.45 in the S.45 to S.8 comparison with GH6 overexpressed in the S.45 to S.35 comparison. The majority of the DEG GHs had a signal peptide and were predicted to be either periplasmic or membrane-anchored (GH3, GH5-Subf33, GH6, GH7, GH28, GH79, GH89, and GH141). Among those with known functions, GH3 is involved in the cleavage or digestion of polysaccharides and GH28 is specific for the metabolism of endogenous glycogen and for pectin catabolism.

There are 1094 genes predicted to be transporters in the CCMP2097 genome; 27 DEGs associated with inorganic ion transport and metabolism and 11 as other transporters were overexpressed under the different conditions (Figs. 5 and 6). Only seven DEGs had a signal peptide and were mainly overexpressed at S.8. Among those, were genes involved with K^+^ transport (PF00520, PF01699, and PF00999, Fig. 5 and Supplementary Table 10) and one ammonium transporter (PF00909). The chloride channel family (PF00654) was only overexpressed at the lowest salinity. One gene, associated with the Na^+^-Pi symporter (PF01384) was progressively overexpressed at each salinity decrease. Nine genes coding for K + channel functions were overexpressed, with five overexpressed at the lowest salinity and four at the highest salinity. For “other transporters”, a betaine–carnitine–choline transporter (BCCT, PF02028), which transports osmolytes during osmoregulation, was overexpressed at the S.45. Of the remaining 21 DEGs, 11 were involved in “lipid transport and metabolism”, 4 in “secondary metabolite biosynthesis, transport and catabolism” and 6 implicated in “intracellular trafficking, secretion, and vesicular transport” (Figs. 5 and 6). Three of the lipid transport and metabolism genes were overexpressed at the highest salinity, among them, one gene was annotated as a fatty acid desaturase (PF00487) and another as a triglyceride lipase (PF01764).

CCMP2097 genome has in total 20 backbone genes coding for secondary metabolites, among the DEGs was a polyketide synthase, annotated as a zinc-binding dehydrogenase (PF00107)^28^, which was overexpressed at S.8. In the secretion category, two DEGs had a signal peptide with one DEG involved in glycosaminoglycan degradation (PF05089) and the other annotated as a clathrin-coat-binding protein with a SET domain (PF00856). Two other DEGs are associated with the formation of synaptic vesicles and secretory granule exocytosis (KOG1028).

Overall, in all nine selected pathways, there were 55 (ca. 62%) unique DEGS, mostly expressed at the S.8 (Fig. 6b), with secondary metabolites and secretion pathways only overexpressed at S.8 (Fig. 6a). Seven DEGs were shared among the three groupings of high (S.35 and S.45), medium (S.16 and S.25), and low salinities (S.8). The 7 genes included: two TRs, a response regulator receiver domain (PF00072) and a SET domain (PF00856); one PK, PKC-CK2 (PF12796); two inorganic ion transporters, a K^+^ channel ERG (PF00520) and a Na^+^–Pi symporter (PF01384); one sugar transporter (PF00083) and one BCCT, an osmolyte transporter (PF02028).

### Antifreeze protein genes

**Fig. 7:**
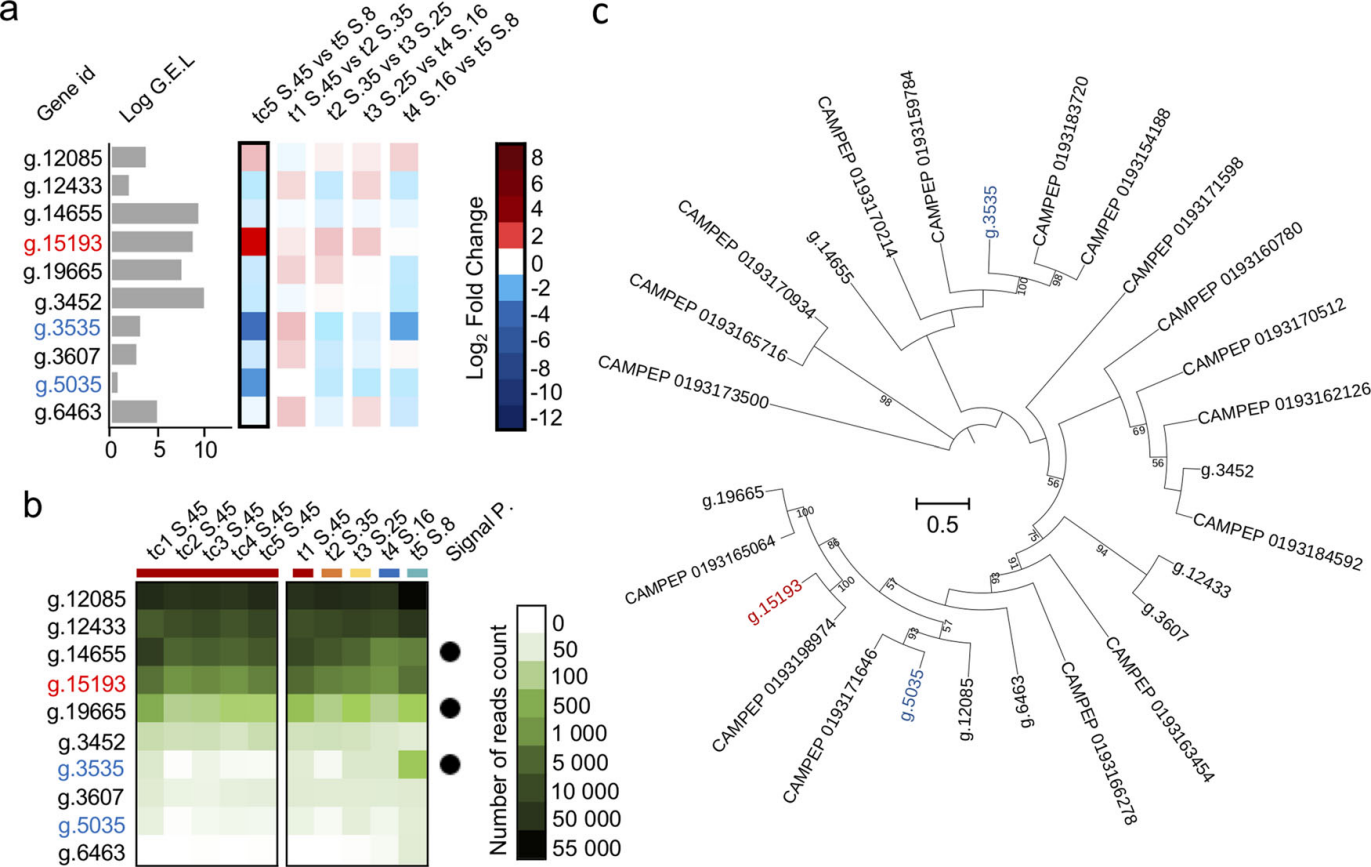
Ice-binding proteins from DUF3494 in this study. **a** Barplot and heatmap of the ten genes coding for the DUF3494 (PF11999). Barplot indicates the log gene expression level (G.E.L.) for each gene. The log_2_ fold change for each comparison of salinity (see Figs. 3 and 4) are indicated by the blue and red colors. **b** Heatmaps show the average (triplicate) of the number of reads per gene for each condition of salinity. On the right, are the control (salinity 45) following the time of sampling. On the left are the results of the progressive change of salinity (S.). The presence of a signal peptide (P.) is indicated with a black circle. **c** Maximum Likelihood tree generated using RAxML PROTGAMMAGTR from an alignment of 27 short sequences from the ten genes coding for ice-binding proteins found in all transcriptomes of this study and 17 short sequences from MMETSP transcriptomes of the same alga (CCMP2097). The tree was constructed with an alignment of 613 amino acids with bootstrap support repeated 1000 times. Only bootstrap support >50 (of 100) is shown.

As Arctic sea-ice microalgae are exposed to freezing conditions, we further investigated protein DUF3494 that is reported to be an IBP^29^. The CCMP2097 genome contains ten genes in this domain (PF11999) and three of these were overexpressed over the experiment (Figs. 4 and 7). Two of the DUF3494 DEGs had a signal peptide (Fig. 7a, b). A RAxML tree constructed with protein sequences from the CCMP2097 genome, and transcripts from the MMETSP dataset^30^ showed the three IBPs, were phylogenetically distant from each other, suggesting alternative mechanisms for protection from freezing at the two most divergent salinities (Fig. 7c).

## Discussion

To respond to external changes or stresses, cells modulate the transcriptome to maintain the internal environment of the cell. If a stress condition persists, cells can acclimate or adjust to the new normal and maintain growth. In the ice-influenced arctic environment, marine microalgae are routinely exposed to salinities both higher and lower than seawater. Here, we found multiple DEGs that are usually associated with stress response, especially salinity but also multiple unknown genes that were differentially regulated as salinity was progressively lowered.

Different IBPs were overexpressed in the pelagophyte at hypo- and hypersaline conditions. Both significant IBPs were associated with a signal peptide suggesting that they would be secreted, conferring protection by external binding of ice (Figs. 4 and 7). IBPs specific to sea-ice diatoms provide a means to moderate their immediate environment within ice^31^ and DUF3494 in sea-ice dinoflagellates is consistent with the IBP as an adaptation to cold^32^. The protein is hypothesized to be either anchored at the outer cell surface or becomes concentrated when cells or cysts form aggregations^33^. Our results suggest that this IBP may also have a crucial role for the adaptation to salinity changes, this may not be surprising given that the freezing temperature of water is determined by salinity, with fresher water freezing more rapidly than saline waters. Another potential key gene, overexpressed at higher salinities, an acyl-esterase involved in freezing resistance/cold acclimation^27^, can modify cell-surface biopolymers^34^ consistent with a signal P and export to the cell surface. Overall, the excretion of some IBPs and antifreeze proteins could ameliorate the negative effect of salt stress by remodeling the extracellular environment, which is a trait of sea-ice diatoms as well^31^.

There are numerous stress-responsive genes regulated by a network of TFs and TRs that are key regulatory components of abiotic stress signaling pathways^35^, and specifically TFs and TRs play crucial roles in salt tolerance in many higher plants^36^. TFs and TRs DEGS in the pelagophyte were implicated in salinity adjustment to both higher and lower salinity. For example, two different Myc TFs were overexpressed, one at the higher salinity (PF00010 (S.45) and the other (PF00176) at S.8, suggesting two opposing functions (Fig. 5). The Myc-type with helix–loop–helix domain (PF00010), in rice can confer salt tolerance and induce the expression of a specific protein to regulate the ratio between inorganic Na^+^ and K^+^ ions used for osmotic regulation^37^. The use of different Myc-types is consistent with the expression of different genes involved inorganic ion transportation being differentially expressed at high and low salinities (Fig. 5).

Under high salinity, several TRs with a response regulator membrane receiver domain (PF00072) were overexpressed. This specific domain is part of a bacterial two-component system^38^ and in higher plants is involved in stress response^39^. In contrast, under low salinity, we report several TRs with a conserved SET domain (PF00856) that is involved in plant defense in response to different environmental stresses^40^ and has an important role in the regulation of histone methylation^41^. Overall, TFs and TRs can indirectly activate a signaling cascade of a whole network of genes that act together to increase stress tolerance in plants under harsh environmental conditions^42^. However, as few of the putative TFs and TRs in our data were identified with known functions, further studies are needed to determine whether the changes in transcript abundance of these putative TFs and TRs are genuinely part of salinity stress responses.

Overexpression of kinases is a frequent reaction to perturbation within the cell, such as disease or stress^43^. Some PKs associated with osmoregulation and salt stress, such as phosphatidylinositol-4-phosphate 5-kinase (PIP5k) are involved in root growth adaptation to hyperosmotic conditions in *Arabidopsis*^44^. In our case, PIP5k was overexpressed at S.8, along with numerous other PKs suggesting a more general stress response (Figs. 5 and 6). In contrast, the 5’AMP-activated PK overexpressed in the pelagophyte under high salt is involved in energy production and conversion; acting as an energy sensor and when activated restores energy homeostasis^45^. It also can inhibit lipid production and control glucose metabolism^46^. This suggests that under the higher salt conditions the pelagophyte was not stressed^47^. The lower lipid production and secretion strategy were also evident, with several triglyceride lipase and fatty acid desaturase genes overexpressed at high salinity and none of the overexpressed genes at high salinity involved in secretion (Fig. 5). On the contrary, when the pelagophyte was exposed to low salt, several specific Ca^2+^-dependent PK (PKC-C2, PF00168) involved in phospholipid binding were overexpressed allowing secretion. In addition, PKC types control growth and cellular differentiation^43^ and PKs activity depends on secondary messengers such as cyclic adenosine monophosphate (AMP) and calcium^43^. For example, the involvement of Ca^2+^-dependent PK in the activation of intracellular signaling was shown in the common ice plant *Mesembryanthenum crystallinum* exposed to high salinities^48^. PKs can also regulate gene transcription by targeting specific TFs, however identifying direct PKs and TFs–TRs relationships involved in a particular pathway is difficult due to the pleiotropic functions of both key proteins and their large spectrum of interactions^49^.

The GH families of CAZymes have different roles in hydrolyzing or rearranging glycosidic bonds and convert polysaccharides to sugars (Figs. 5 and 6 and Supplementary Table 9). Two GHs (GH7 and GH28) are associated with salt tolerance^50,51^. The overexpression of CAZymes under low salt stress suggest that the pelagophyte could metabolize lipids and polysaccharides as an energy source. The cell wall is the primary defense for an organism to acclimate or adapt to an environmental stress, consequently structural changes in cell walls aid survival by providing a physical barrier and preventing the release of defense signaling molecules^50,52^. Wetherbee et al.^14^ described an extracellular perforated theca in sand-dwelling pelagophytes and hypothesized that it provided structure and strength and could protect cells against salinity fluctuations. Pelagophyte cell walls contain alginate and cellulose^53,54^ and the GHs overexpressed under low salt, included GHs families that degrade, modify or create glycosidic bonds^55^, suggesting additional mechanisms for adaptation to salinity fluctuation.

Salinity is intricate stress that can influence varied physiological and biochemical pathways. Osmoregulation in cyanobacteria exposed to higher salinities is a three-step process. They first adjust their turgor pressure by transporting water into the cell, then adjust ion concentrations and finally synthesize osmolytes^56^. Osmolytes are highly soluble small organic molecules^57^, also known as compatible solutes and are crucial for the cell to adapt to hyper- or hypoosmotic stress. At higher external salinities most organisms, like cyanobacteria, regulate ion concentrations using the “salt out” strategy keeping low internal inorganic ion concentrations and lowering the water potential, which is aided by the accumulation of osmolytes^58^. Osmolytes serve mainly to compensate for the difference in water potential, which help cells to take up water under high salinity or expel water under low salinity to maintain turgor pressure^56^. At the higher salinity, our pelagophyte employed a version of a similar “salt out” strategy, where several K^+^ transporters are overexpressed at high compared to low salinities (Fig. 5). K^+^ is a key monovalent cation inside the cell and is preferentially accumulated inside with the exchange of the toxic Na^+^^56^.

While the cell actively exports ions under hypersaline conditions, to keep internal ion concentrations constant during hyposaline conditions cells import ions^59^. Here, several Na^+^ transporters were overexpressed at the lower salinity. Na^+^ is the main cation in oceans and is the main danger when it enters the cells under hypersaline conditions, the overexpression of symporter (PF01384), antiporter (PF00999) and exchanger (PF01699) of Na^+^ under low salt suggest Na^+^ is required for homeostasis in the pelagophyte. Moreover, we found aquaporin DEGs overexpressed at S.8. Aquaporins, act by adjusting the flux of water (Figs. 4–6), and although importing water, is crucial when cells are exposed to higher salinity^60^, in this case exporting water would have been a primary function of the aquaporins at S.8.

Under the influence of osmotic stress, microalgae can survive by modifying their lipid content^61^, mainly triacylglycerols^62^ as energy stores that becomes available when cells encounter favorable conditions^63^. However, not all microalgae use this strategy of lipid accumulation, and some synthesize secondary metabolites from primary metabolites such as lipids, carbohydrates, and amino acids^64,65^. Here, this strategy resulted in the production of secondary metabolites under low salt (Figs. 5 and 6). In addition, the overexpression of several genes coding for acyl-CoA synthase, and DEGs involved in lipid transporters would serve to secrete lipids (Fig. 5), suggesting another role for lipid metabolism for coping with low salinity.

In keeping with our starting hypothesis, we found that TFs, TRs, PKs, CAZymes, and transporters were differentially expressed at high compared to low salinities. DEGs involved in osmotic regulation showed increasing overexpression of Na^+^–H^+^ antiporters and Na^+^–Pi symporters as salinity decreased, but the overexpression of genes in the K^+^ channel complex at higher salinities. We found the potential implication of IBPs and antifreeze proteins with a differential expression suggesting tight coupling between cold temperature adaptation and salinity, in keeping with higher salinities lowering the freezing point of water. Such adaptations are consistent with a recent global ocean interactome study of plankton by Chaffron et al.^66^, which found that Arctic planktonic communities are fundamentally different from temperate and tropical marine communities. The pelagophyte used for this experiment has only ever been found in the Arctic^22^ adding weight to the notion that Arctic marine microbial eukaryotes may be genetically primed to be adapted to or at least able to cope with the broad range of salinities found in Arctic surface waters^67^. Our transcriptional approach identified genes that were expressed following acclimation to salinity changes over a modest (not instant or evolutionary) time frame.

## Methods

### Sample collection

The Arctic pelagophyte CCMP2097 was initially collected from a small ice melt pocket on the surface of the ice from Northern Baffin Bay in June 1998 and isolated using a serial selection–dilution technique until a unialgal species was confirmed with light microscopy and 18S rRNA Sanger sequencing. The isolate was maintained in culture in L1^68^ media made up in aged 0.2 µm-filtered seawater collected between 300 and 500 m from the North Water region, with a salinity of 33–35. Cultures were maintained at ca. 4 °C as described in Terrado et al.^69^ and under constant illumination of 100 µmol photon m^−2^ s^−1^, which is near the average PAR levels for the summer surface waters at the latitude where the culture originated^70^.

### Salinity change cultures and nucleic acid isolation

For the experiment we used a 0.22 µm-filtered artificial seawater from the Laboratoire aquatique de recherche en sciences environnementales et médicales (LARSEM), Université Laval, Québec, QC, Canada). Salinity was measured with an EcoSense™ EC300A Conductivity Meter (YSI Inc.) and adjusted by dilutions to obtain the target range of salinity from 45, 35, 25, 16 to 8. The experimental and control cultures were transferred to the highest salinity of 45 for initial acclimation based on a scenario of the spring community of algae growing in brine channels at around that salinity^5^. Cells were grown in L1 medium (National Center for Marine Algae and Microbiota, NCMA) with added antibiotics for 18 days until the exponential phase, dilution media also contained antibiotics. We collected 200 mL from the culture, when cells were at late exponential phase and added 200 mL of new medium with a lower salinity to obtain a culture with a salinity of 35 (Supplementary Fig. 1). We let the cells grow ca. 5 days until a second harvest at the late exponential phase. Every ca. 5 days, we harvested cells at the lower salinity and added increasing amounts of freshwater media until the final salinity of 8, cells did not grow in freshwater. For a control experiment, we grew cells in the same incubator conditions, but always at the salinity of 45. We sampled cells at the same time as the salinity treatments and added new medium without changing the salinity until the end of the experiment. The aim of this control at the same salinity, was to monitor any time or dilution effects on the cultures. The Percival incubator was maintained at 4 °C throughout the experiment with 100 µmol photon m^−2^ s^−1^.

Each triplicate of culture for each condition was sampled at late exponential phase following Terrado et al.^69^. We harvested cells directly from the individual flasks after gentle mixing. To collect cells directly from culture flasks we used sterile 60-mL syringes (BD, Canada) (Supplementary Fig. 1). For RNA, the full 60 mL of the culture were then immediately filtered through a 0.22-µm pore size Sterivex™ Unit (Millipore™ Canada Ltd.). The Sterivex™ filters were then flash-frozen in liquid nitrogen and stored at −80 °C.

### Cellular viability, nutrients consumption, and pigment concentration

At the time of each harvest, we sampled 2 mL of each culture flask to evaluate the proportion of live and dead cells. Each sample was stained with Invitrogen SYTOX™ Green Nucleic Acid Stain (ThermoFisher Scientific) to differentiate live and dead cells^71^ and enumerated using BD Accuri™ C6 Flow Cytometer (BD Biosciences) equipped with the Csampler and 14.7 mW 640 nm Diode Red Laser and 20 mW 488 nm Solid State Blue Laser. Live and dead cells were separated based on its relative size using forward scattered light (FSC) and relative chlorophyll green fluorescence intensity (FL1) at 530 nm (Supplementary Fig. 17). Data acquisition was performed at a slow flow rate (46 µL min^–1^) with three agitation and wash cycles between each sample and recalibrated at each time of sampling with 2 μm Fluoresbrite™ beads (BD Trucount™) in filtered seawater to normalized cells counts with flow rate.

To determine nutrient consumption by cells at the time of the harvest, we filtered the media collected using a 60-mL syringe through 25 mm Swinnex™ filter holder with a glass fiber filter (Whatman™ GF/F, No. 1825-025). The filtrate was collected into Sarstedt™ 15 mL conical tubes and then frozen at −80 °C. Nutrient concentrations were measured using an Autoanalyzer 3 (Bran and Luebe) with standard colorimetric methods adapted from Grasshof et al.^72^.

To determine major pigment concentration of cells, we collected 20 mL from the cultures at each salinity condition. The pigment samples were collected by syringe filtration onto with a Whatman GF/F 25-mm filter. These filters were wrapped in aluminum foil and stored at −80 °C until analysis. We then prepared samples and measured the pigments using Thermo Scientific system (Thermo Scientific, West Palm Beach, FL, United States) fitted with a Hypersil Gold C8 High-Performance Liquid Chromatography (HPLC) column (3-µm pore size, 4.6 × 150 mm, Thermo Scientific). Briefly, pigments were extracted from frozen filters by sonication (three times for 20 s at 17 W intensity) in 95% MeOH, and then filtered using a PTFE 0.2-µm filter^73^. Analyses of pigments were performed by injecting samples filtrates to the HPLC system and pigments were detected by Photodiode Array and fluorescence spectroscopy^73^.

### Nucleic acid extraction, library preparation, and sequencing

RNA of cells was extracted from the Sterivex™ Units using the RNeasy™ mini kit (Qiagen™). The quality and the quantification of RNA content were verified using Thermo Scientific™ NanoDrop™ 8000 spectrophotometer (Fisher Scientific™) as well as Qubit fluorometer (Life Technologies™) using the RNA BR Assay Kit (Invitrogen™). Samples passing the quality checks were sent to the Department of Energy (DOE) Joint Genome Institute (JGI) in Walnut Creek, California for RNA quantity and quality verification, followed by library preparation and sequencing. Plate-based RNA sample preparation was performed on the PerkinElmer Sciclone™ NGS robotic liquid handling system using Illumina’s TruSeq™ Stranded mRNA HT sample prep kit utilizing poly-A selection of mRNA, following the protocol outlined by Illumina in their user guide. The following conditions were applied: total RNA starting material was 1 µg per sample and eight cycles of PCR was used for library amplification. The prepared libraries were quantified using KAPA Biosystems’ next-generation sequencing library qPCR kit and run on a Roche LightCycler™ 480 real-time PCR instrument. Sequencing of the flowcell was performed on the Illumina NovaSeq™ sequencer using NovaSeq™ XP V1 reagent kits, S4 flowcell, following a 2 ×150 indexed run recipe. For comparison sequence data was processed using two independent approaches.

### Sequence data processing at DOE JGI

Using BBDuk (https://sourceforge.net/projects/bbmap/), raw reads were evaluated for artifact sequence by kmer matching (kmer = 25), allowing 1 mismatch and detected artifact was trimmed from the 3’ end of the reads. RNA spike-in reads, PhiX reads and reads containing any Ns were removed. Quality trimming was performed using the phred trimming method set at Q6. Finally, following trimming (minimum length 36 pb), reads under the length threshold were removed (minimum length 25 bases or 1/3 of the original read length—whichever is longer). Filtered reads from each library were aligned to the reference genome^24^ using HISAT2 (v. 2.1.0). Raw gene counts calculated with featureCounts^74^ were used to evaluate the level of correlation between biological replicates using Pearson’s correlation and determine which replicates would be used in the differential gene expression analysis using *DESeq2* (v.1.18.1) to determine which genes were differentially expressed between pairs of conditions. A *P* value < 0.05 was used to call a gene DE between conditions.

### Sequence data processing with a customized method

Clean reads from DOE JGI were visualized using FastQC (v.0.11.9) and then trimmed using Trimmomatic v.0.36 (paired-end mode; minimum length 100 pb), this would result in less than 100% gene mapping. The CCMP2097 reference genome^24^ was retrieved from JGI PhycoCosm Portal. Filtered transcriptome reads were mapped onto the genome with STAR (v.2.7.8a) using default parameters. All further analyses were performed using RStudio (v.1.4.1106). The differential gene expression analyses were done using *DESeq2* packages in R. All transcriptomes were normalized using *DESeq2* packages, where counts are divided by sample-specific size factors determined by the median ratio of gene counts^75^. Functional annotation was done by retrieving the annotation available on the JGI Genome Portal. We performed the transcription factors prediction among up and downregulated gene lists using iTAK database (v1.6) and PlanTFDB database (v5.0; http://planttfdb.cbi.pku.edu.cn/). We used the iTAK database to predict protein kinases, and for carbohydrate-active enzymes we used the CAZY database. Consistent with other studies, the two methods to identify DEGs found similar results^76^.

### Calculation

To calculate the growth rate per day, we divided the total number of cells per mL measured by flow cytometry at the time of sampling by the number of cells at the start of the culture until harvest for each batch, multiplied to the 1/N power and subtracting one. N represents the number of days between the initial and final concentrations of cells. To determine the consumption of nutrients by cells, we calculated the initial concentration added at the start of the culture and subtracted to the final concentration measured at the time of sampling. To calculate the Chl *a* per cell, we converted µg L^−1^ to pg cell^−1^ to evaluate the health of cells exposed to the different conditions. We calculated the concentration of major pigments in µmol L^−1^ per µmol of Chl *a*.

### Weighted gene co-expression network analysis (WGCNA) of time and salinity change

To further analyze the time effect and the progressive change of salinities among cultures we performed WGCNA using *WGCNA* package in R. The adjacency matrix between different genes was constructed with a soft thresholding power of 18 for only control samples (*n* = 14) and with a soft thresholding power of 16 for all samples (*n* = 28). All significant DEGs were hierarchically clustered based on topological overlap matrix (TOM) similarity and performed by Dynamic Hybrid Tree Cut and modules with annotated colors defined as branches from the tree cutting. Modules with fewer than 50 genes were merged into their closest larger neighbor module. In addition, the correlations between modules and with the change of salinity, time of sampling, and culture of triplicate were investigated using the Pearson correlation to find key modules associated with these parameters.

### Statistics and reproducibility

Two-way analysis of variance (ANOVA) was conducted to analyze the time effect between salinity change and control conditions on nutrient concentration (two replicates for each triplicate, *n* = 6), on the cellular viability (triplicate, *n* = 3), and on growth rate (triplicate, *n* = 3) using software Past3 (v.3.18). Principal Component Analysis (PCA) and dendrograms were computed using *rda()* and *hclust()* functions in R from *vegan* and *stats* packages respectively, to separate the transcriptome samples of each condition, and between triplicates. The dendrogram was constructed using the complete linkage method which finds similar clusters. Constrained Correspondence Analysis (CCA) was computed using *cca()* function, to discriminate different transcriptomes for each salinity condition according to the nutrient consumption values and the real salinity values measured at the time of sampling. To represent the up- and downregulated gene with differential gene expression relative to the log-fold change, we used visualization through scatter (MA) plots and volcano plots computed with *DESeq2* package. We filtered the list of up- and downregulated genes of each comparison of salinity conditions based on the adjusted *P* value of <0.05. Supplementary Fig. 1 was created entirely using Microsoft® PowerPoint v16.60.

### Reporting summary

Further information on research design is available in the Nature Research Reporting Summary linked to this article.

## Supplementary information

Supplementary Information

Reporting Summary

## Data Availability

All transcriptome samples are in the DOE JGI Genome Portal under Sequencing Project ID 1253386 and Analysis Project ID 123385, from the Sequence Read Archive (SRP284677-SRP284681). CCMP2097 reference genome and the annotated genome are available at JGI Genome Portal and PhycoCosm Portal under JGI Project ID 1020062.

## Code availability

Computer codes and scripts are available at https://osf.io/h73f8/.
