# Supplementary material for: Salinity tolerance mechanisms of an Arctic Pelagophyte using comparative transcriptomic and gene expression analysis
Source: Commun Biol. 2022 May 25;5:500. doi: 10.1038/s42003-022-03461-2 (PMC9133084; doi:10.1038/s42003-022-03461-2)
Supplement: Supplementary file 1 — Supplementary Information [file 42003_2022_3461_MOESM1_ESM.pdf]

Salinity tolerance mechanisms of an Arctic Pelagophyte using comparative transcriptomic and gene expression analysis

Nastasia J. Freyria[1,2*], Alan Kuo[3], Mansi Chovatia[3], Jenifer Johnson[3], Anna Lipzen[3], Kerrie W. Barry[3], Igor V. Grigoriev[3,4] and Connie Lovejoy[1,2*]

[1]Département de biologie, Institut de Biologie Intégrative et des Systèmes, Université Laval, Québec, Canada

[2]Québec Océan, Département de biologie, Université Laval, Québec, Canada

[3]U.S. Department of Energy Joint Genome Institute, Lawrence Berkeley National Laboratory, Berkeley, CA 94720, USA

[4]Department of Plant and Microbial Biology, University of California Berkeley, Berkeley, CA 94720, USA

*Corresponding Authors

Supplementary Table 1. Cellular counts and cellular viability from flow cytometry with the use of Invitrogen SYTOX™ Green Nucleic Acid Stain (see Supplementary Fig. 2a-b). Salinity measured at the time of sampling for each triplicate and concentration of chlorophyll *a* (Chl *a*). Asterix corresponds to the two samples that couldn't be sequenced with RNA-seq.

**Samples**			**Cellular concentration**				
		**Salinity measured**	**Live cells (Cell.ml^{-1})**	**%**	**Dead cells (Cell.ml^{-1})**	**%**	**Chl *a* (pg.cell^{-1})**
Salinity change							
t1 S. 45	1	46.4	1.40E+06	91.6	1.27E+05	8.4	0.026
	2	44.9	1.17E+06	87.2	1.72E+05	12.8	0.021
	3	46.7	1.15E+06	87.2	1.69E+05	12.8	0.020
t2 S. 35	1	35.5	1.94E+06	99	1.94E+04	1	0.024
	2	35.3	1.33E+06	89.5	1.56E+05	10.5	0.034
	3	36.0	1.36E+06	86.5	2.12E+05	13.5	0.010
t3 S. 25	1	24.5	1.60E+06	99.6	5.13E+03	0.4	0.032
	2	24.1	1.38E+06	97.8	3.11E+04	3.2	0.022
	3	24.6	1.00E+06	88.9	1.24E+05	11.1	0.045
t4 S. 16	1	15.2	2.57E+06	61.8	1.59E+06	38.2	0.018
	2	15.8	2.60E+06	80.7	6.23E+05	19.3	0.021
	3	16.0	2.12E+06	68.7	9.65E+05	31.3	0.023
t5 S. 8	1	8.0	2.64E+06	97.1	7.77E+04	2.9	0.028
	2	7.9	2.08E+06	77.3	6.08E+05	22.7	0.035
	3*	8.1	4.34E+05	18.35	1.93E+06	81.65	0.030
Control							
tc1 S. 45	1*	43.1	4.55E+05	30.7	1.02E+06	69.1	-
	2	46.5	1.23E+06	90.6	1.26E+05	9.4	0.092
	3	46.3	5.88E+05	33.6	1.16E+06	66.4	-
tc2 S. 45	1	44.4	5.72E+05	89.3	6.81E+04	10.7	-
	2	40.3	9.83E+05	87.2	1.43E+05	12.8	0.041
	3	40.1	7.86E+05	85.3	1.35E+05	14.7	-
tc3 S. 45	1	45.1	7.80E+05	62.8	4.62E+05	37.2	-
	2	47	1.23E+06	75.8	3.92E+05	24.2	0.050
	3	47.9	8.59E+05	59	5.96E+05	41	-
tc4 S. 45	1	44.5	1.74E+06	94.8	9.49E+05	5.2	-
	2	46.5	2.02E+06	93.3	1.44E+05	6.7	0.023
	3	46.4	1.25E+06	64.3	6.92E+05	35.7	-
tc5 S. 45	1	46.1	2.66E+06	99.69	8.03E+03	0.31	-
	2	46.5	2.25E+06	99.5	1.05E+04	0.5	0..027
	3	45.7	2.52E+06	98.47	3.91E+04	1.53	-

Supplementary Table 2. Mean nutrient concentrations (duplicate of each triplicate) at time of sampling and nutrients consumptions from calculation explained in methods (see Supplementary Fig. 2c-d).

		Nutrient concentration (μmol.L^{-1})		Nutrient consumption (μmol.L^{-1})	
**Sample**	**Triplicate**	**Nitrite+Nitrate**	**Phosphate**	**Nitrite+Nitrate**	**Phosphate**
Salinity change					
t1 S. 45	1	821.30	22.09	61.17	14.11
	2	481.85	12.09	400.47	24.11
	3	585.41	12.04	296.80	24.16
t2 S. 35	1	556.61	7.27	705.62	32.92
	2	393.31	5.83	529.68	24.35
	3	740.80	10.79	286.11	19.35
t3 S. 25	1	340.45	2.08	657.08	23.29
	2	284.03	3.80	550.48	20.13
	3	502.54	2.50	678.98	26.38
t4 S. 16	1	179.85	1.09	601.47	19.09
	2	225.38	0.79	499.32	21.11
	3	322.91	0.53	620.47	20.07
t5 S. 8	1	319.09	0.24	301.67	18.95
	2	321.90	0.34	344.34	18.55
	3	351.29	1.08	412.72	17.55
Control					
tc1 S. 45	1	506.60	11.11	375.85	25.09
	2	613.13	15.34	269.19	20.86
	3	644.47	14.70	237.86	21.50
tc2 S. 45	1	415.26	8.52	532.57	20.68
	2	503.41	8.28	550.90	25.15
	3	504.82	7.35	580.88	25.45
tc3 S. 45	1	321.45	1.96	534.72	24.66
	2	294.99	1.47	649.60	24.91
	3	319.90	1.76	625.99	23.69
tc4 S. 45	1	215.03	0.71	547.66	19.35
	2	149.64	0.93	586.41	18.64
	3	167.35	0.52	593.57	19.34
tc5 S. 45	1	132.52	1.65	524.19	17.16
	2	95.48	1.41	495.45	17.62
	3	85.54	1.45	522.99	17.17

Supplementary Table 3. Statistic two-way ANOVA for concentration of total nitrate and total phosphate consumed by cells (n = 12, two replicates for each triplicate) for both control and for salinity change groups (see Supplementary Fig. 2c-d). Total degree of freedom of 23.

	Repeated measures ANOVA					
	Nitrite+Nitrate			Phosphate		
**Time of sampling**	**F**	***p*-value**	**Significance**	**F**	***p*-value**	**Significance**
Time 1 vs time 2	1.05	0.48		0.42	0.70	
Time 2 vs time 3	0.21	0.82		0.19	0.83	
Time 3 vs time 4	0.42	0.70		0.58	0.63	
Time 4 vs time 5	19.26	**0.04**	*	0.58	0.62	
Time 1 vs time 5	0.20	0.82		0.18	0.84	

* Significant *p*-values.

Supplementary Table 4. Pigments content in μmol.L^{-1} normalized by μmol of Chl *a* for each condition of salinity (S.) of each triplicate of progressive change of salinity and for only one triplicate of control. The corresponding abbreviations mentioned in Supplementary Fig. 3 are given in the first column. Time of sampling are given as t1 to t5 for progressive salinity change and as tc1 to tc5 for control (see Supplementary Fig. 1).

		**Concentration of pigment in μmol.$^{-1}$ per µmol Chl *a***																			
		**Triplicate number**															**Control (S. 45)**				
		**t1 S. 45**			**t2 S. 35**			**t3 S. 25**			**t4 S. 16**			**t5 S. 8**			**tc1**	**tc1**	**tc1**	**tc1**	**tc1**
**Abrreviation**	**Name**	**1**	**2**	**3**	**1**	**2**	**3**	**1**	**2**	**3**	**1**	**2**	**3**	**1**	**2**	**3**	**2**	**2**	**2**	**2**	**2**
19-but-fuco	19-but-fucoxanthin	0.192	0.183	0.196	0.207	0.176	0.195	0.198	0.164	0.218	0.158	0.176	0.182	0.166	0.148	0.145	0.020	0.195	0.196	0.163	0.169
B,B-caro	B,B-carotene	0.000	0.003	0.002	0.003	0.002	0.000	0.002	0.004	0.000	0.000	0.003	0.003	0.003	0.003	0.002	0.000	0.001	0.004	0.002	0.004
Chl c2	Chl c2	1.338	1.261	0.460	1.250	1.222	1.014	1.122	1.086	1.126	0.980	1.091	1.071	0.870	0.814	0.818	0.111	0.947	1.190	1.075	1.143
Chl c2-like	Chl c2-like	0.000	0.020	0.000	0.012	0.019	0.014	0.013	0.008	0.014	0.017	0.010	0.013	0.006	0.027	0.026	0.002	0.073	0.027	0.074	0.047
Chlide a	Chlorophyllide a	0.095	0.071	0.073	0.056	0.065	0.066	0.077	0.078	0.077	0.062	0.062	0.054	0.043	0.040	0.033	0.013	0.100	0.105	0.112	0.112
Diadinochr.	Diadinochrome-like	0.016	0.026	0.045	0.039	0.035	0.027	0.034	0.029	0.034	0.035	0.031	0.030	0.029	0.022	0.020	0.002	0.002	0.040	0.025	0.029
Dd	Diadinoxanthin	0.914	0.930	0.728	1.389	1.241	1.062	1.515	1.452	1.162	1.333	1.500	1.170	1.320	1.164	1.032	0.071	0.012	1.252	1.234	1.403
Dt	Diatoxanthin	0.568	0.621	0.524	0.436	0.510	0.290	0.653	0.628	0.560	0.000	0.549	0.483	0.422	0.383	0.360	0.030	0.480	0.705	0.531	0.608
Fuco	Fucoxanthin	4.179	3.914	4.172	3.924	3.831	3.413	3.482	3.409	3.480	2.988	3.320	3.215	2.617	2.469	2.490	0.364	3.140	3.650	3.372	3.573
Gd	Gyroxanthin dodec-ethano-like	0.024	0.025	0.019	0.019	0.020	0.019	0.019	0.021	0.018	0.005	0.020	0.019	0.016	0.015	0.012	0.002	0.017	0.020	0.016	0.016
Myxo	Myxoxanthophyll	0.018	0.011	0.011	0.007	0.006	0.010	0.007	0.012	0.010	0.007	0.009	0.008	0.008	0.006	0.006	0.001	0.010	0.008	0.009	0.004
Pheo a	Pheophytin a	0.112	0.181	0.164	0.151	0.302	0.107	0.142	0.288	0.154	0.236	0.143	0.132	0.107	0.216	0.188	0.016	0.137	0.136	0.165	0.164
Vauch	Vaucheriaxanthin-like	0.008	0.007	0.000	0.003	0.007	0.004	0.005	0.002	0.004	0.005	0.002	0.005	0.005	0.007	0.006	0.001	0.014	0.008	0.010	0.008
Viola	Violaxanthin	0.059	0.035	0.097	0.035	0.022	0.041	0.013	0.009	0.026	0.009	0.010	0.011	0.007	0.007	0.007	0.007	0.063	0.018	0.009	0.010
Zea	Zeaxanthin	0.010	0.019	0.005	0.007	0.018	0.016	0.012	0.016	0.012	0.013	0.013	0.012	0.016	0.022	0.022	0.001	0.026	0.014	0.025	0.020

Supplementary Table 5. Overall results of Illumina transcriptome sequencing.

Sample	Triplicate	QC% reads	% of reads > Q30 average	Raw data (reads)	Trimmed data (reads)	Mapping coverage (%)
Salinity change						
t1 S. 45	1	62.93±5.06	96.06	10,477,783	9,796,771	90.48
	2	62.49±6.41	96.03	41,379,778	39,378,979	89.98
	3	62.08±5.93	96.03	34,887,172	32,985,926	90.95
t2 S. 35	1	63.18±6.19	95.80	23,751,407	22,116,330	91.36
	2	61.88±6.61	96.12	50,715,031	47,063,079	93.27
	3	62.45±5.67	96.12	10,950,740	10,345,004	91.15
t3 S. 25	1	64.12±5.62	95.49	32,080,191	30,380,472	92.53
	2	63.42±5.14	96.14	23,843,525	22,375,892	91.36
	3	63.21±5.17	96.09	32,665,975	30,508,265	92.14
t4 S. 16	1	62.94±5.43	95.93	15,170,137	14,275,179	92.15
	2	63.23±5.70	95.29	24,566,804	23,315,594	92.79
	3	61.80±5.64	95.92	33,282,898	31,398,684	92.14
t5 S. 8	1	62.77±5.92	95.81	30,446,944	28,991,878	92.85
	2	62.86±5.59	95.77	35,914,835	34,187,558	92.36
Control						
tc1 S. 45	2	62.81±5.87	95.99	45,479,076	41,951,985	89.23
	3	63.78±5.41	95.73	13,923,610	13,008,865	91.01
tc2 S. 45	1	63.68±5.46	95.16	25,182,361	23,574,241	92.61
	2	62.48±5.29	96.37	29,658,195	28,049,338	88.56
	3	63.10±6.22	95.36	26,016,402	24,663,074	90.12
tc3 S. 45	1	64.41±5.22	95.32	38,324,449	36,114,378	92.38
	2	63.41±5.93	95.80	33,010,291	31,219,185	93.49
	3	62.77±6.09	96.08	19,963,517	18,457,711	91.98
tc4 S. 45	1	62.88±5.67	95.84	13,342,803	12,453,557	92.21
	2	62.99±5.37	95.27	40,988,288	38,324,279	92.75
	3	62.93±5.47	95.15	15,348,638	14,358,258	89.23
tc5 S. 45	1	64.36±5.11	94.77	38,091,163	35,115,434	91.38
	2	62.85±5.99	95.44	17,617,208	16,395,079	91.95
	3	63.00±5.52	95.03	38,896,449	35,061,127	92.49

Supplementary Table 6. Comparison between the two methods used (DOE JGI method and the customized method) for analyzing differential genes expression. The total number of differentially expressed genes (DEGs) are indicated for each method. Unique DEG retrieved for each method and the number of shared DEGs between the two methods.

		**DOE JGI method**		**Customized method**		**Number of shared DEG**
		Total number of DEG	Number of unique DEG	Total number of DEG	Number of unique DEG	
Salinity change						
t1 S.45 vs t2 S.35	Up	0	0	4	4	0
	Down	4	4	14	14	0
t2 S.35 vs t3 S.25	Up	3	0	19	16	3
	Down	8	1	36	29	7
t3 S.25 vs t4 S.16	Up	1	0	18	17	1
	Down	8	0	19	11	8
t4 S.16 vs t5 S.8	Up	10	0	27	17	10
	Down	42	5	66	29	42
t1 S.45 vs t5 S.8	Up	75	4	184	113	75
	Down	319	6	685	372	319
Control						
tc1 S.45 vs tc2 S.45	Up	3	1	20	18	2
	Down	0	0	26	26	0
tc2 S.45 vs tc3 S.45	Up	0	0	0	0	0
	Down	0	0	1	1	0
tc3 S.45 vs tc4 S.45	Up	0	0	0	0	0
	Down	2	0	4	2	2
tc4 S.45 vs tc5 S.45	Up	0	0	0	0	0
	Down	1	1	1	1	0

Supplementary Table 7. Annotation of differential expressed genes of opposite sense shared among comparisons of change of salinities from Fig. 3c.

Conditions when DEGs were shared	Gene ID	Functional annotation
Up t4 S.16 and Down t2 S.35	12610	PF02872, 5'-nucleotidase C-terminal domain (Apyrase family)
Up t3 S.25 and Down t2 S.35	13193	PF12796, Ankyrin repeats
Up t4 S.16 and Down t1 S.45	13306	Unknown
Up t1 S.45 and Down t4 S.16	13818	SSF54001
Up t3 S.25 and Down t1 S.45	14038	PR01217, Proline rich extensin signature 4
Up t3 S.25 and Down t1 S.45	14400	Unknown
Up t4 S.16 and Down t1 S.45	15872	PF02492, cobalamin biosynthesis CobW-like, C-terminal
Up t3 S.25, Down t2 S.35 and Down t4 S.16	16707	PF02338, OTU-like cysteine protease
Up t2 S.35 and Down t1 S.45	16915	Unknown
Up t3 S.25, Down t2 S.35 and Down t4 S.16	16941	Unknown
Up t2 S.35 and Down t4 S.16	17122	PF07452, Chordin domain,
Up t3 S.25, Down t1 S.45 and Down t4 S.16	1803	PF02492, cobalamin biosynthesis CobW-like, C-terminal
Up t2 S.35 and Down t1 S.45	18792	PF12796, Ankyrin repeats
Up t3 S.25 and Down t4 S.16	19428	Unknown
Up t3 S.25 and Down t2 S.35	19774	Unknown
Up t2 S.35 and Down t1 S.45	4396	Unknown
Up t2 S.35 and Down t1 S.45	4411	PF14259, RNA recognition motif (RRM, RBD, or RNP domain)
Up t4 S.16 and Down t4 S.16	4425	Unknown
Up t2 S.35 and Down t1 S.45	4430	PF07671, Protein of unknown function (DUF1601)
Up t2 S.35, Down t1 S.45 and Down t3 S.25	4764	Unknown
Up t2 S.35 and Down t1 S.45	5319	PF00027, Cyclic nucleotide-binding domain (cAMP/cGMP motif)
Up t1 S.45 and Down t2 S.35	5540	PF08238, Extracellular protein SEL-1
Up t1 S.45 and Down t3 S.25	5702	PF00487, Fatty acid desaturase
Up t2 S.35, Down t1 S.45 and Down t3 S.25	6424	Unknown
Up t2 S.35 and Down t1 S.45	7958	Unknown

Supplementary Table 8. Number of filtered (F.) up- and down-regulated genes (with adjusted p-value of < 0.05) annotated with InterProScan, GO terms, KOG and KEGG pathways, genes with Signal peptide (Sig. P.) and unknown genes (Unk.) between each comparison of each condition of salinity.

	Number of genes							
**Condition**	**Total F.**	**Annotated**	**Unk.**	**KEGG**	**KOG**	**IPR**	**GO**	**Sig. P.**
**Upregulated genes**								
tc1 S.45 vs tc2 S.45	20	12	8	2	6	13	4	2
tc2 S.45 vs tc3 S.45	0	0	0	0	0	0	0	0
tc3 S.45 vs tc4 S.45	0	0	0	0	0	0	0	0
tc4 S.45 vs tc5 S.45	0	0	0	0	0	0	0	0
t1 S.45 vs t2 S.35 (up 45)	4	3	1	0	0	2	1	1
t2 S.35 vs t3 S.25 (up 35)	19	15	4	1	0	11	3	11
t3 S.25 vs t4 S.16 (up 25)	18	11	7	1	1	7	3	9
t4 S.16 vs t5 S.8 (up 16)	27	21	6	3	13	16	14	10
tc5 S.45 vs t5 S.8 (up 45)	158	85	73	9	32	84	39	44
**Downregulated genes**								
tc1 S.45 vs tc2 S.45	26	13	13	0	6	12	14	3
tc2 S.45 vs tc3 S.45	1	1	0	0	0	1	1	0
tc3 S.45 vs tc4 S.45	4	2	2	0	0	1	0	1
tc4 S.45 vs tc5 S.45	1	1	0	0	1	1	1	0
t1 S.45 vs t2 S.35 (down 35)	14	10	4	3	6	7	4	3
t2 S.35 vs t3 S.25 (down 25)	36	30	6	7	16	28	15	7
t3 S.25 vs t4 S.16 (down 16)	19	16	3	4	5	13	6	8
t4 S.16 vs t5 S.8 (down 8)	66	51	15	14	27	47	25	14
tc5 S.45 vs t5 S.8 (down 8)	501	266	235	69	158	259	148	111

Supplementary Table 9. The classification and characteristic features of carbohydrate active enzymes Glycoside Hydrolases (GHs) from transcriptomes of CCMP2097 of only up- and down-regulated genes list. Information is based on iTak database and PlantTFDB database (v.5.0).

**Family**	**Clan**	**Mechanism**	**Activity in plant**	**Putative function**
Glycoside hydrolase (GH)				
GH3	A	Retaining	β-xylosidase, β-glucosidase, α-L-arabinofuranosidase, β-N-acetylhexosaminidase, glucan 1,3-β-glucosidase, glucan 1,4-β-glucosidase, a-L-arabinofuranosidase, and exo-1,3-1,4-glucanase	Cleavage and digestion of polysaccharide
GH5	A	Retaining	β-mannanase and β-glucosidase	Cellulase
GH6	B	Inverting	xyloglucan endotransglycosylase	Cellulobiohydrolase
GH7	C	Retaining	Unknown	Cellulobiohydrolase
GH16	B	Retaining	Xyloglucan endotransglycosylase hydrolase	Cell wall carbohydrate biosynthesis and modification
GH28	N	Inverting	Polygalacturonase	Pectin remodeling and degrading enzymes, cell expansion
GH31	D	Retaining	α-xylosidase, α-glucosidase, and isomaltosyltransferase	Unknown
GH79	A	Retaining	g β-glucoronidase	Unknown
GH89	Unknown	Retaining	α-N-acetylglucosaminidase	Unknown
GH141	Unknown		α-L-fucosidase, xylanase	Pectin remodeling or degrading enzymes

Supplementary Table 10. PFAM, SUPERFAMILY and PROSITE number for each gene differentially expressed found among the nine selected pathways from Fig. 5.

Selected pathways	PFAM, SUPERFAMILY and PROSITE number	Description
Transcription regulator (TR)	PF00505	HMG (high mobility group) box
	PF00072	Response regulator receiver domain
	PF00856	SET domain
	PF00176	SNF2 family, Myc-type domain
	SSF144232	Zinc finger (HIT/MYND type)
	PF00628	Zinc finger, PHD-type
	PF1390	Zinc finger, C3HC4 type (RING finger)
	PF10276	Zinc finger, CHCC type
Transcription factor (TF)	PF13385	ConcanavalinA-like lectin/glucanases
	PF00010	Myc-type (basic helix-loop-helix) domain
	PF01753	Zinc finger (MYND type)
Protein kinase (PK)	PF00571	5’ AMP-activated Kinase (CBS domain)
	PF00069	AGC PKA-PKG CAMK/CDPK
	PF00069	AGC RSK-2 (Ribosomal S6 Kinase 2)
	PF00069	AGC/NDR and related serine/threonine kinase
	PF13405	Ca2+/calmodulin-dependent PK, EF-hand Ca2+ binding
	PF01471	cAMP/cGMP binding motif, Peptidoglycan binding
	PF02493	PIP5K, MORN repeat
	PF12796	PKC-C2, ankyrin
	PF00168	PKC-C2, Ca2+ dependent phospholipid-binding
	PF00481	Protein phosphatase 2C (PP2C)-like domain
	PF07714	Tyrosine kinase specific for activated GTP-bound (TKL Cr-4)
Lipid	PF03982	Acyl-CoA: diacylglycerol acyltransferase (DAGAT)
	PF01425	Amidases, carbon-nitrogen ligase
	PF00650	CRAL-TRIO lipid binding
	PF00378	Enoyl-CoA hydratase/isomerase
	PF00487	Fatty acid desaturase
	PF00005	Lipid exporter ABCA1
	PF00328	Lysosomal and prostatic acid histidine phosphatases
	PF03062	Membrane bound O-acyl transferase (MBOAT)
	PF01734	Patatin-like phospholipase A2
	PF05721	Phytoanoyl-CoA dioxygenase
	PF01764	Triglyeride lipase
Inorganic ion trasnporter	PF00909	Ammonium transporter, ammonia permease
	PF08016	Ca2+ modulated nonslective polycystin channel
	PF00066	Ca2+ modulated nonslective polycystin channel, LNR domain
	PF02010	Ca2+ modulated nonslective polycystin channel, REJ domain
	PF00484	Carbonic anhydrase
	PF00654	Cl- channel
	PF00654	Cl- channel, CLC-7
	PF00122	E1-E2 ATPase, Ca2+ transporting ATPase
	PF00520	K+ channel
	PF02214	K+ channel BTB/POZ domain

	PF00520	K+ channel ERG, PAS/PAC sensor domain
	PF00520	K+ channel KCNQ
	PF02214	K+ channel tetramerization-type BTB domain
	PF01699	K+ channel Ca2+/Na+ exchanger NCKX1
	PF00999	K+/H+ antiporter
	PF00924	Mechanosensitive ion channel MscS
	PF03553	Na+/H+ antiporter
	PF01384	Na+/Pi symporter
	SSF90112	Neurotransmitter-gated ion-channel transmembrane domain
	PF01384	Phosphate transporter
	PF00690	Plasma membrane H+ transporting ATPase
Other transporter	PF00230	Aquaporine
	PF03547	Auxin efflux carrier, membrane transport
	PF02028	Betaine/Carnitine/Choline transporter (BCCT)
	PF02492	Cobalamin (vitamin B12) biosynthesis CoBW-like
	PR00120	Proton pump, H+ transporting ATPase
	PF07690	Major Facilitator family (MFF)
	PF02460	Patched transmembrane receptor, MMPL domain
	PF00083	Sugar transporter (MFF)
	PF07690	Synaptic vesicle transporter
	PF01490	Transmembrane amino acid transporter
	PF03151	Triose-phosphate transporter domain
Secondary metabolites	PF01179	Copper amine oxidase
	PF01794	Ferric reductase, NADH/NADPH oxidase
	PF00107	Polyketide synthase (Zinc-binding dehydrogenase)
Secretion	PF05089	Alpha-N-acetylglucosaminidase
	PF00856	Clathrin coat binding protein/HIP1
	PF13967	Late exocytosis
	PF04096	Nuclear pore complex, Nup98 component
	PF00168	PKC-C2, synaptic vesicle/secretory granule exocytosis
	PF12359	Vesicle coat complex COPII, DUF3645

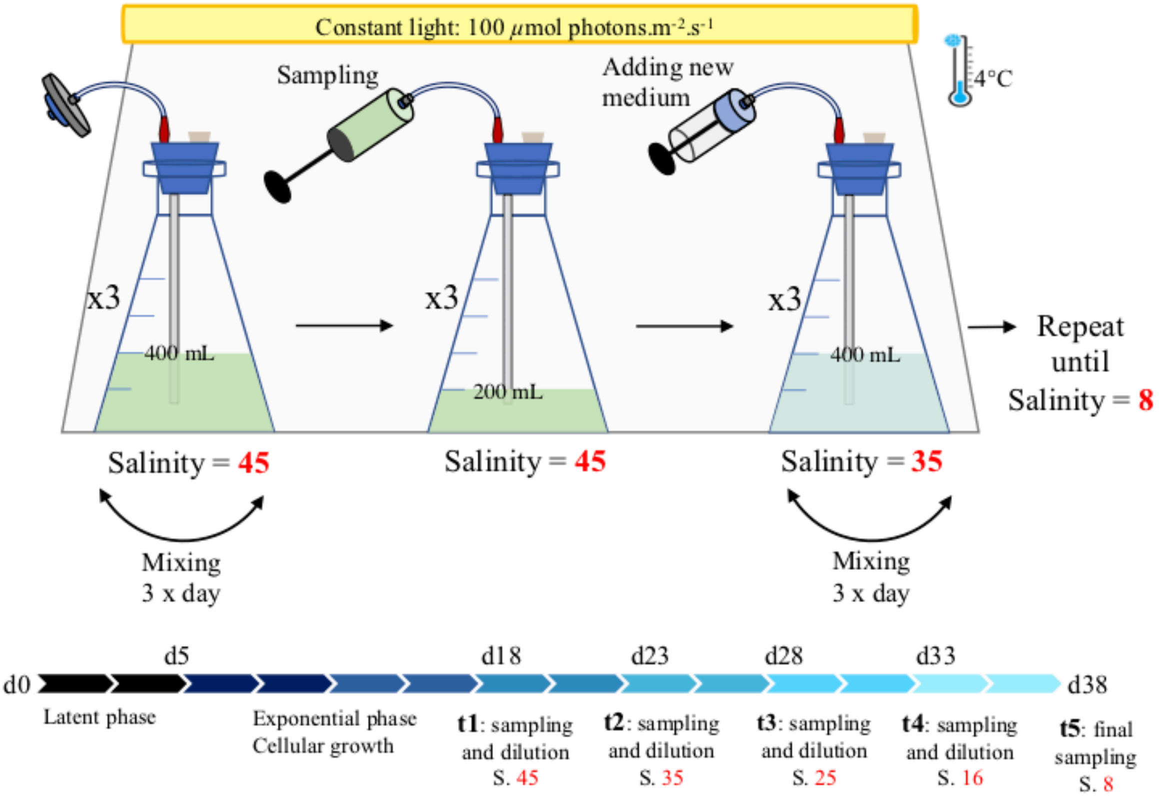

Supplementary Fig. 1. **Experiment design for salinity progressive change**. Number of days of culture are indicate above the chronological scale. Time of sampling is indicated as t1 to t5. For control experiment the salinity was the same all the long with the salinity 45.

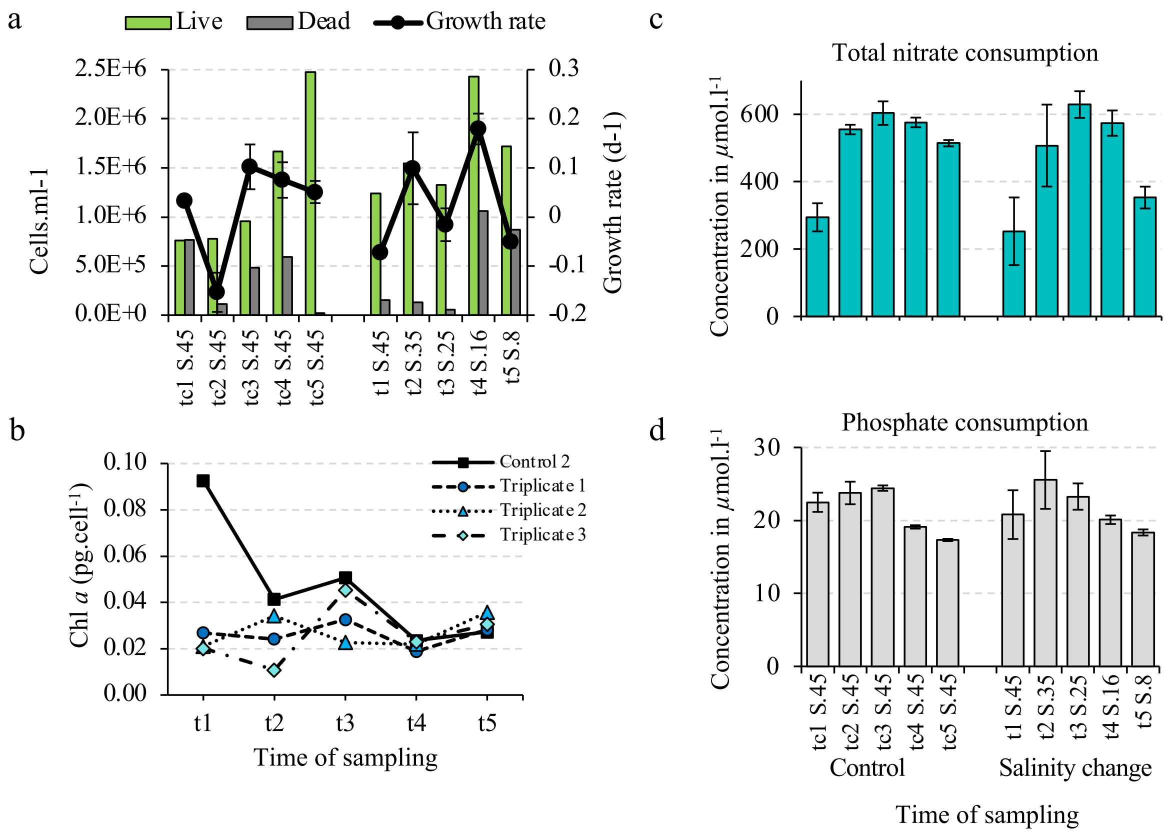

Supplementary Fig. 2. Cellular growth, nutrient consumption and cell health. a. Cellular viability with concentration of alive and dead cells per mL at the time of sampling, and growth rate per day of total cells in each condition. Error bars of growth rate include triplicate values (n = 3). **b.** Concentration of Chl *a* per cell for each triplicate of culture and one triplicate of control. **c.** and **d.** Nutrients (total nitrate and phosphate) consumptions at the time of sampling. Error bars include triplicate culture with biological replicates (n = 6). Standard deviation was used to calculate error bars.

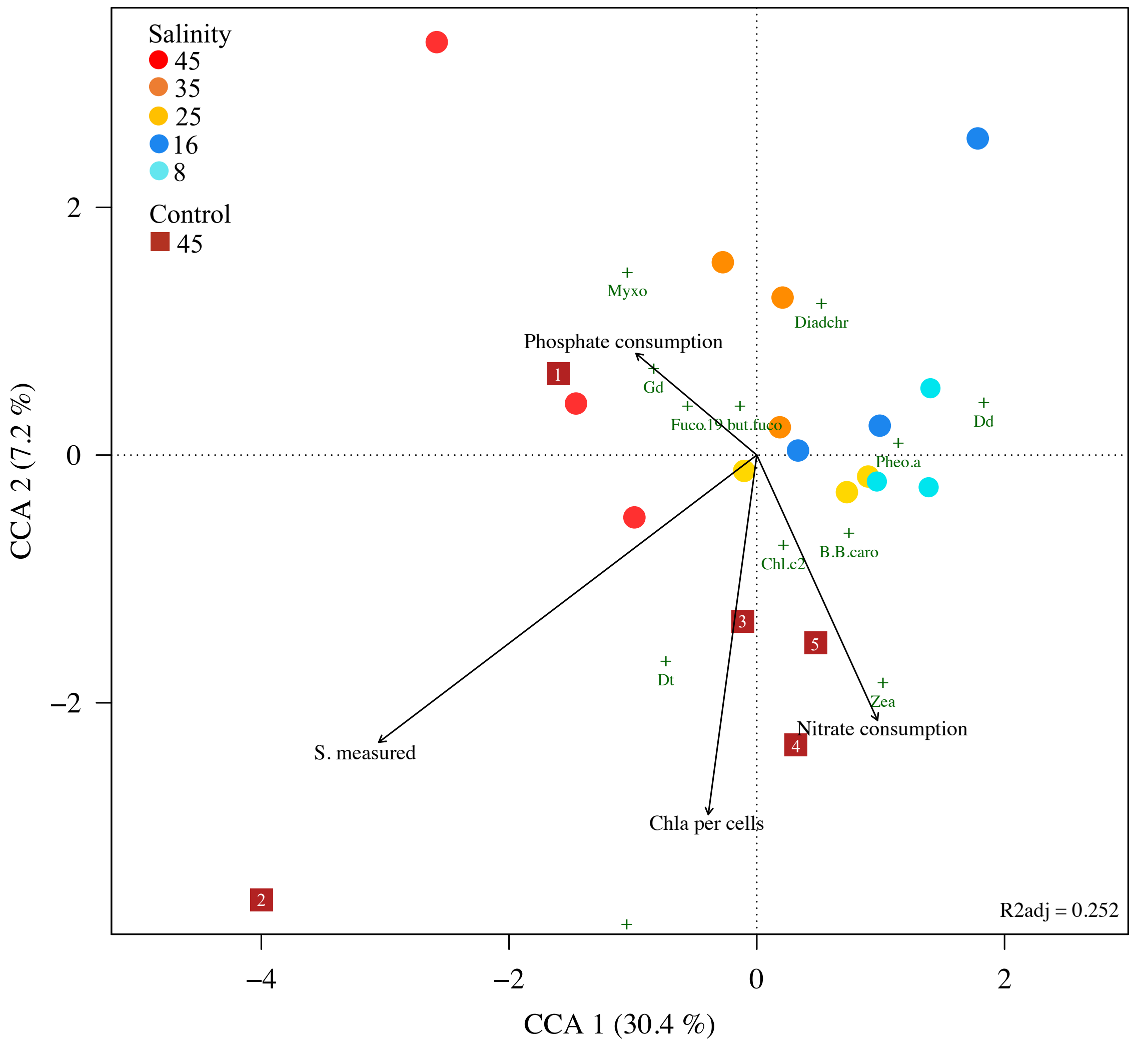

Supplementary Fig. 3. Concentration of pigments per μmol of Chl *a*. Pigments results clustered by Bray-Curtis dissimilarity and a Constrained Correspondence Analysis (CCA). Arrows indicate significant correlations between culture parameters and pigment distribution. Pigments separated following salinity treatments indicated by colored circles along the first axis. Salinity controls (45; dark red squares) were more dispersed. The number in the squares indicate the time of sampling for time control samples. The separation along the second axis suggests the control samples tended to increase chl *a* per cell and nitrate consumption over time.

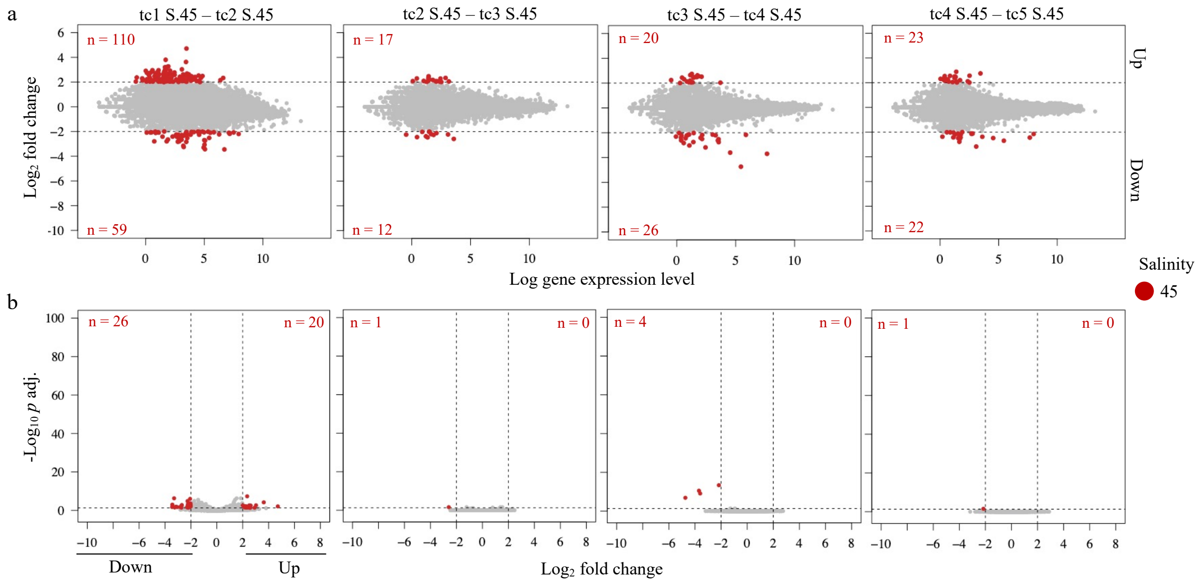

Supplementary Fig. 4. Differential expression genes from control (salinity 45). a. Comparison scatter plot of RNA-Seq analyses of the control group. Each point represents a unigene. Points higher than 2 of $\log_2$ fold change indicate genes up-regulated and each point lower than -2 of $\log_2$ fold change indicate genes down-regulated. Each number (n) of up- and down-regulated genes are indicated. **b.** Scatter plot of filtered up- and down-regulated genes with an adjusted *p* value of < 0.05.

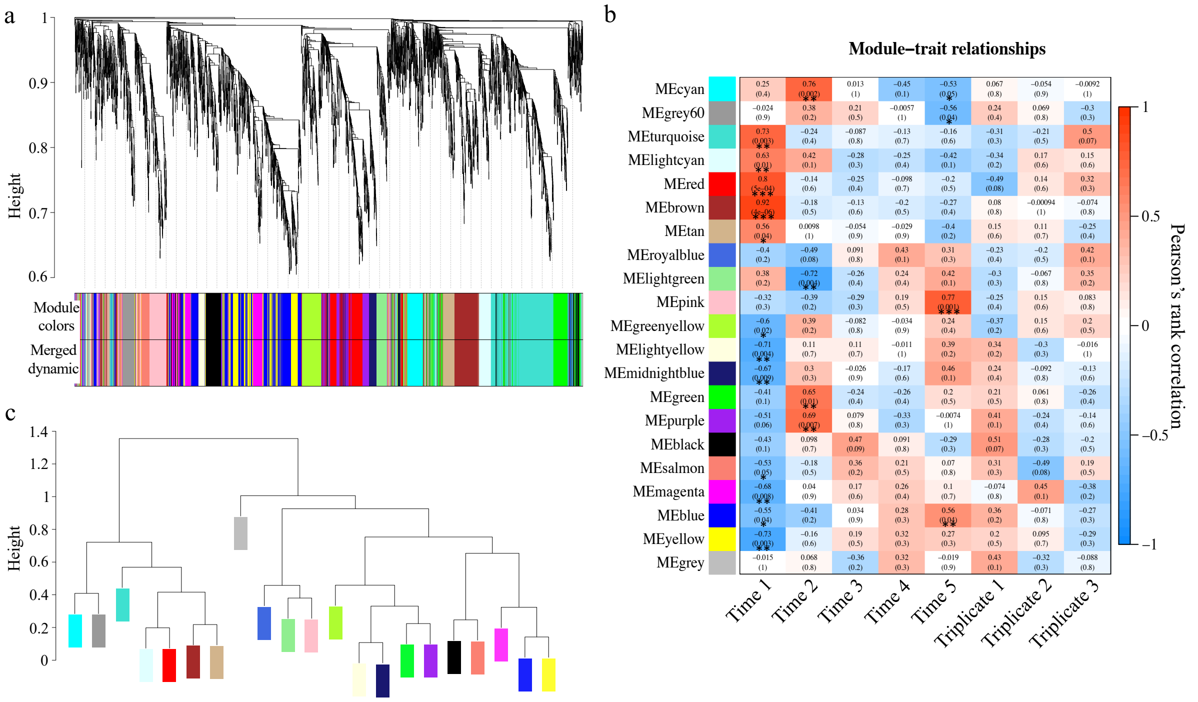

Supplementary Fig. 5. Weighted Gene Co-expression Network Analysis (WGCNA) of control samples. a. A dendrogram plot with color modules annotation and differentially expressed genes were clustered into 21 modules. Genes were clustered based on a dissimilarity measure (1-TOM). **b.** Module with time of sampling, and triplicates weighted correlations and corresponding *p*-values. The color scale showed module-trait correlation based on Pearson's rank correlation. **c.** A module network dendrogram constructed by clustering module eigengene distances. Asterisks within circles represent the significance level *p*-value from Pearson's *Rho* (p <0.05 *, p <0.01 **, p <0.001 ***).

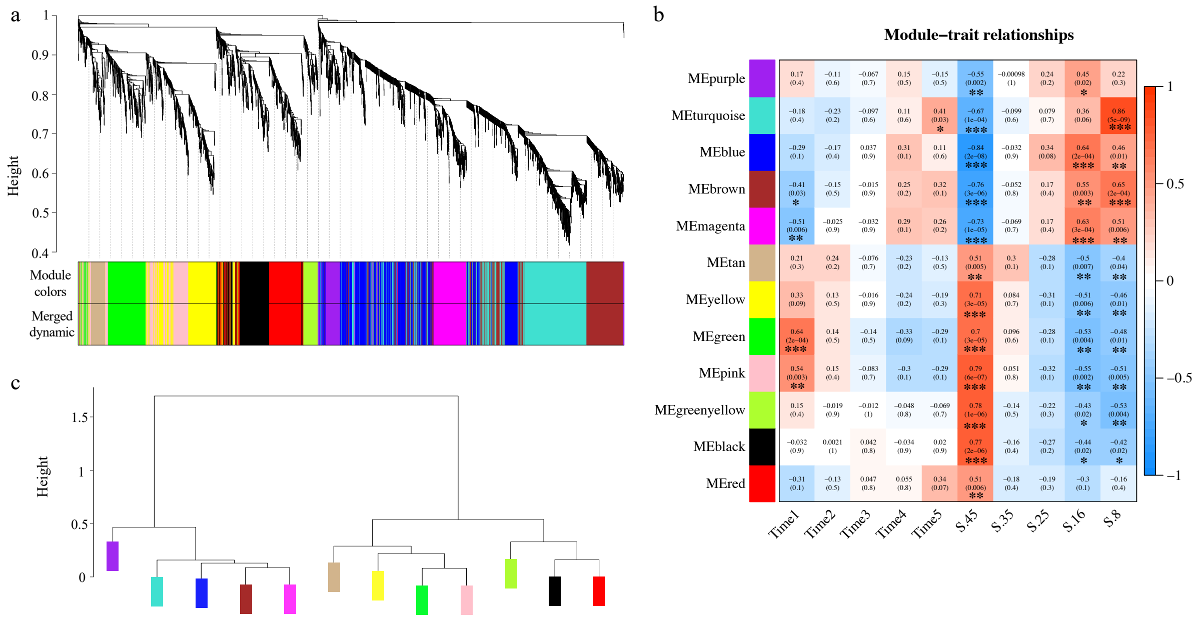

Supplementary Fig. 6. Weighted Gene Co-expression Network Analysis (WGCNA) of all samples. a. dendrogram plot with color modules annotation and differentially expressed genes clustered into 12 modules based on a dissimilarity measure (1-TOM). **b.** Module with time of sampling, salinity change, and triplicates weighted correlations and corresponding *p*- values. The blue to red scale indicates module-trait correlation strength based on Pearson's rank correlation. **c.** Module network dendrogram constructed by clustering module eigengene distances. Asterisks within circles represent the significance level *p*-value from Pearson's *Rho* (*p* <0.05 *, *p* <0.01 **, *p* <0.001 ***).

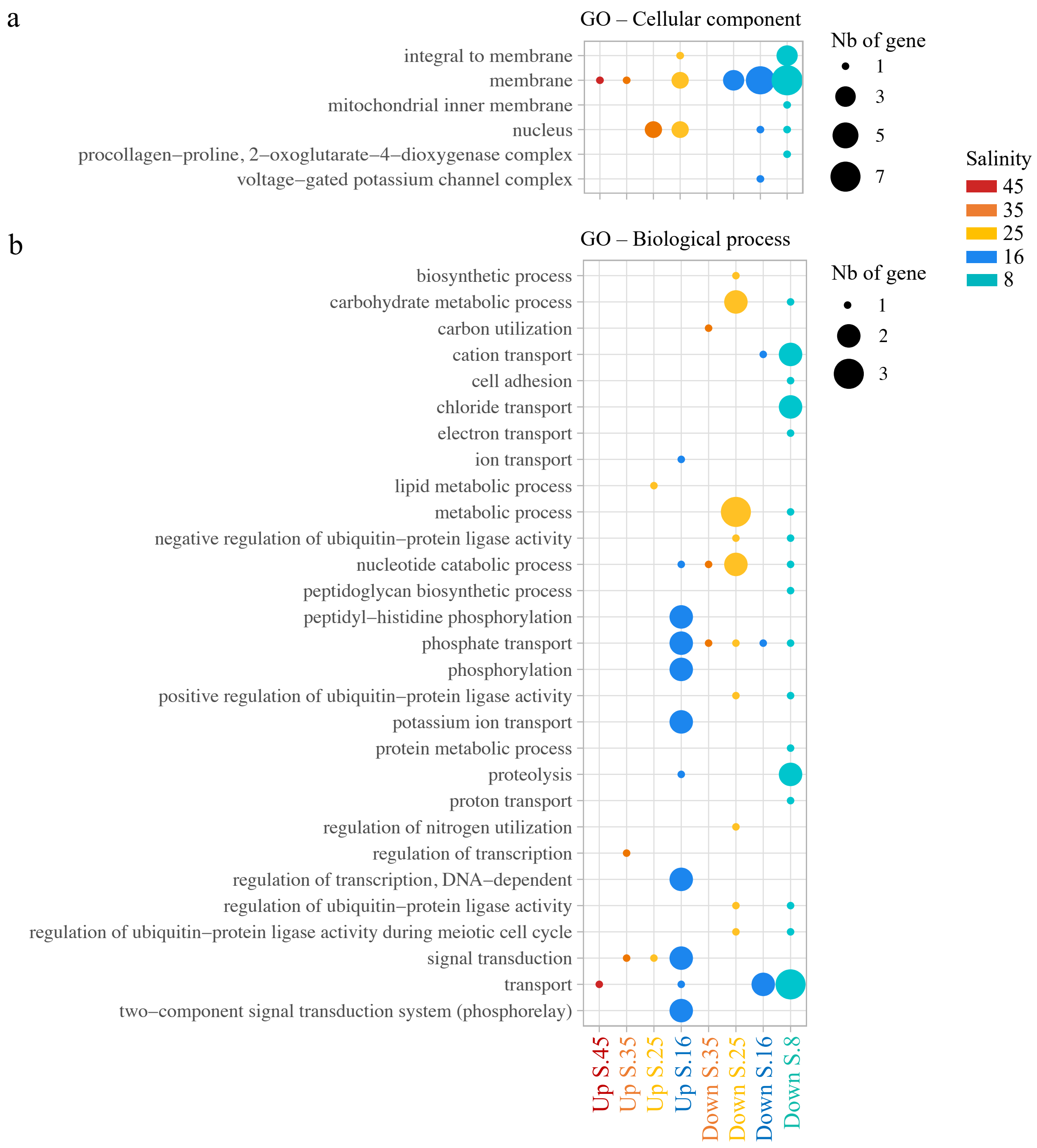

Supplementary Fig. 7. GO term enrichment of cellular component and biological process from progressive change of salinities. Annotations of up- and down-regulated genes for of salinity change (see Fig. 3).

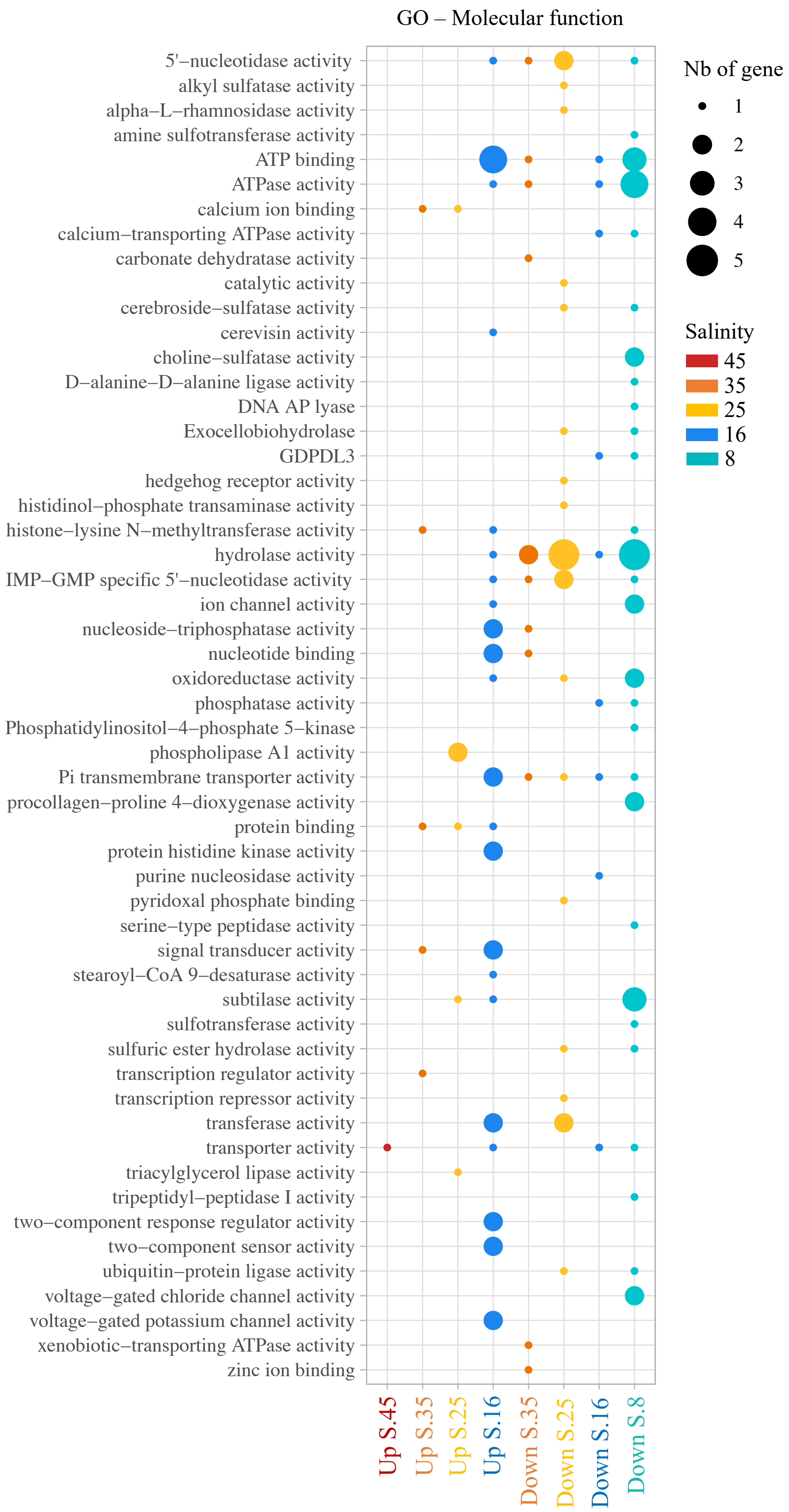

Supplementary Fig. 8. GO term enrichment of molecular function from progressive change of salinities. Annotations of up- and down-regulated genes for each comparison of salinity change (see Fig. 3).

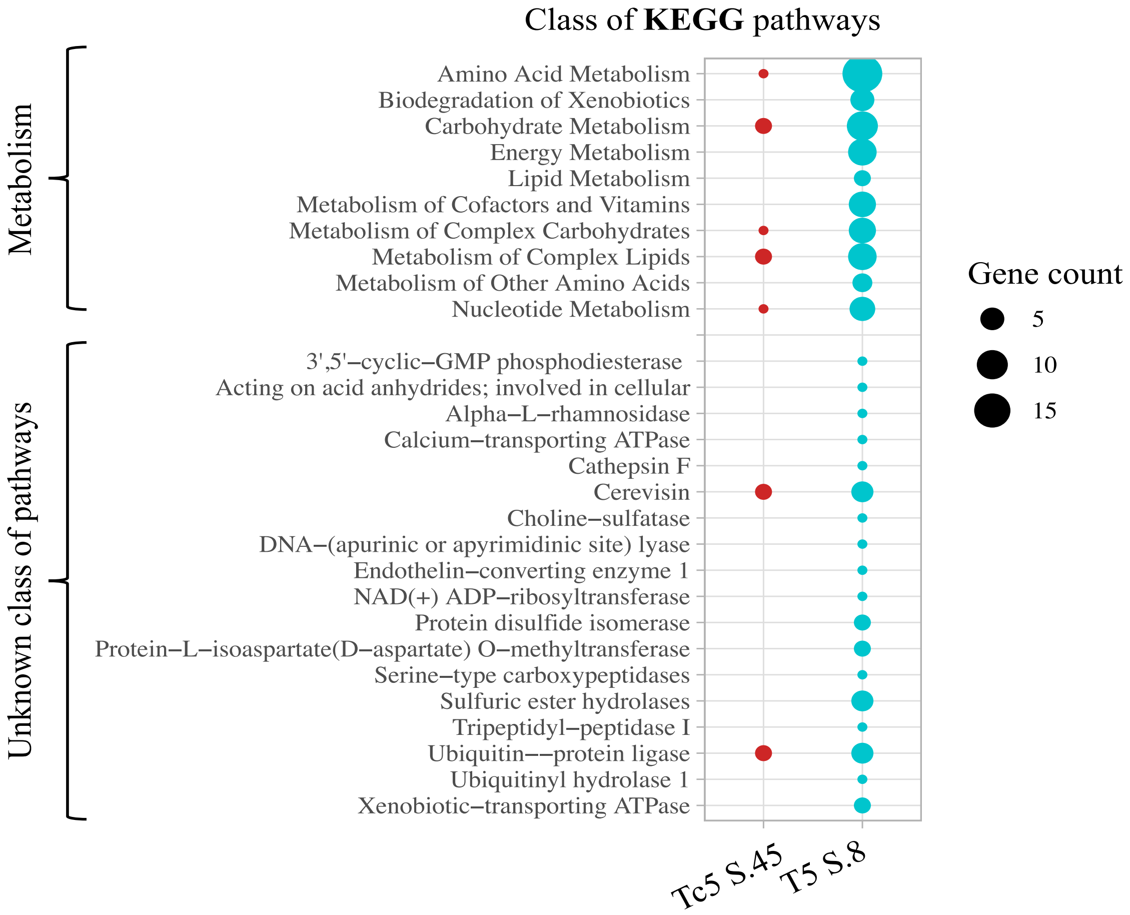

Supplementary Fig. 9. KEGG pathway class from the highest and the lowest salinities (45 versus 8).

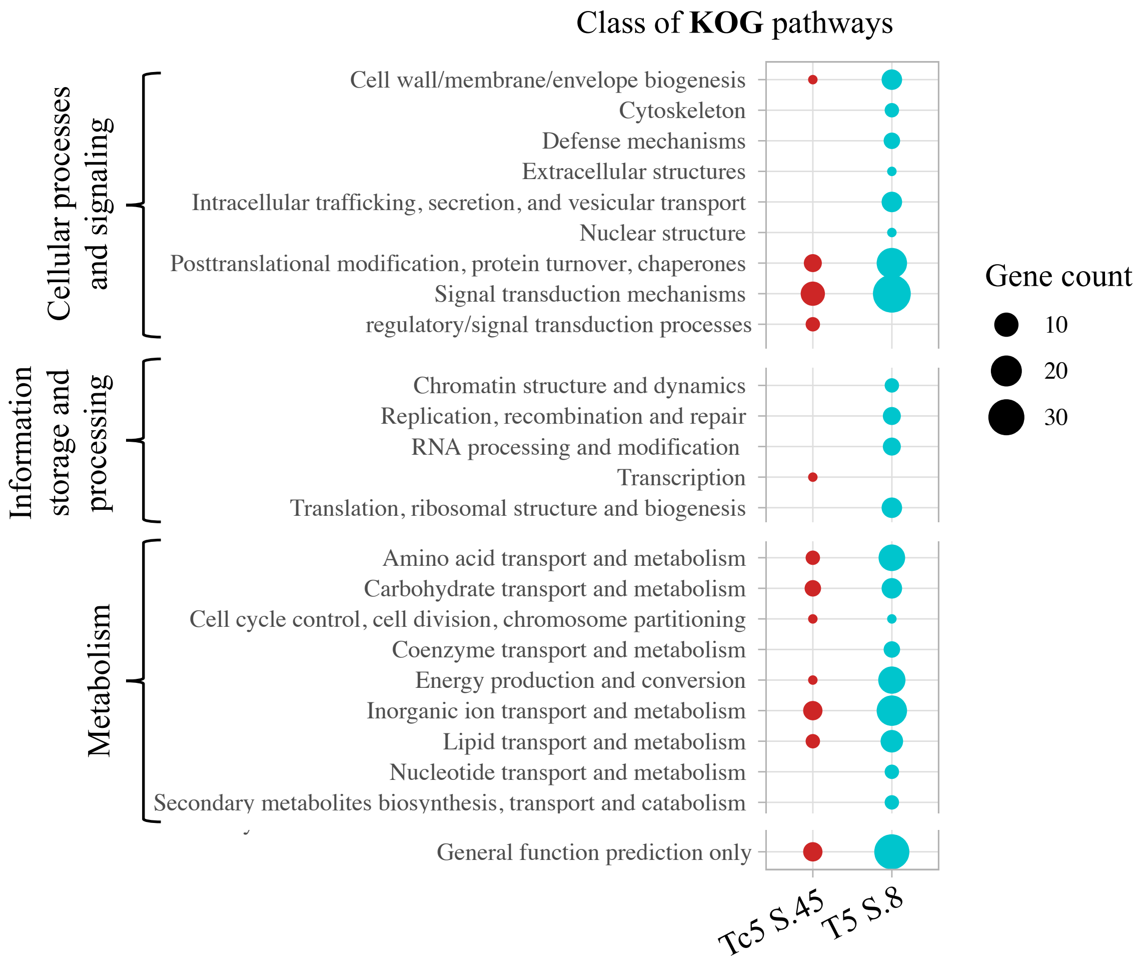

Supplementary Fig. 10. KOG pathway class from the highest and the lowest salinities (45 versus 8).

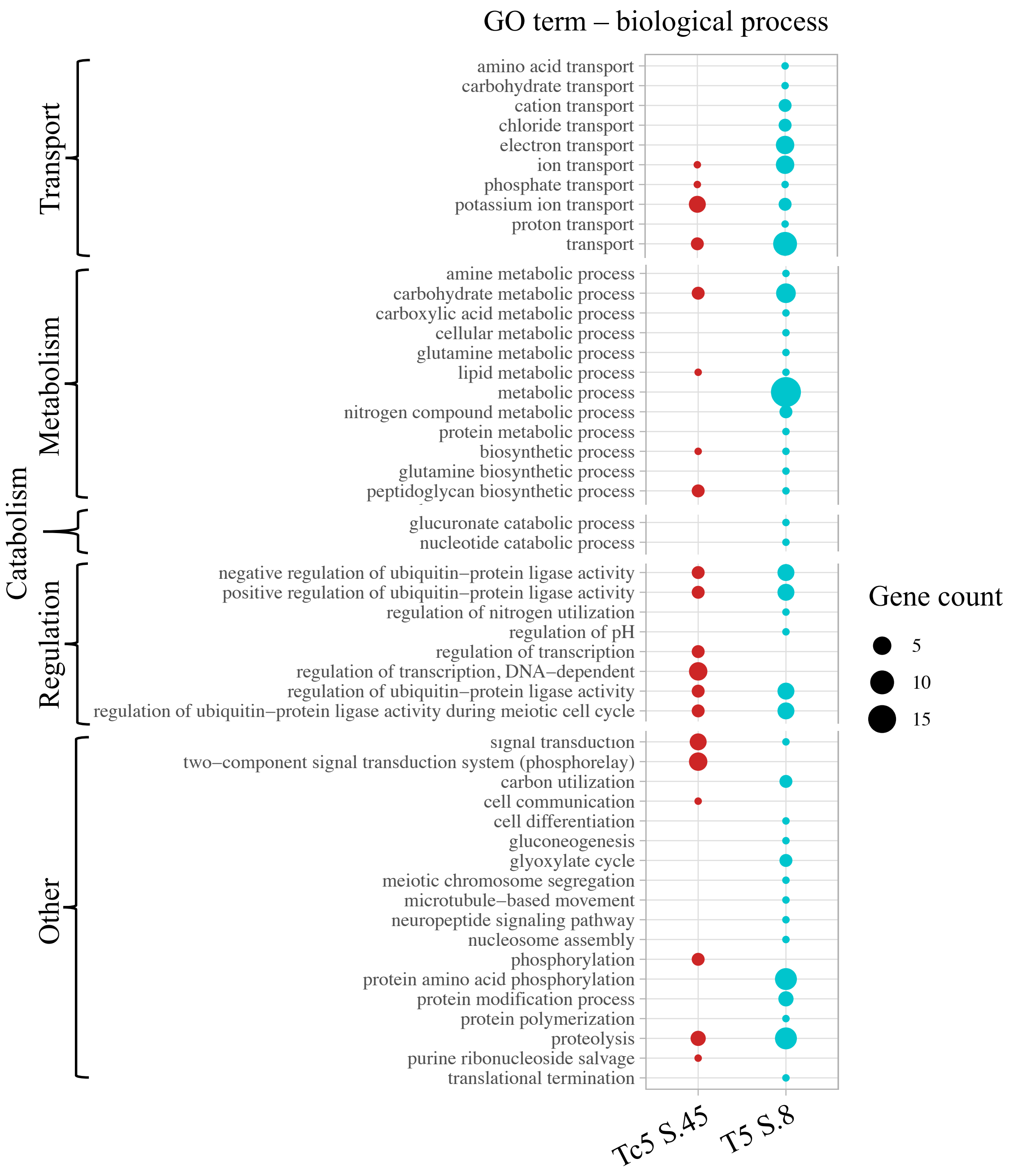

Supplementary Fig. 11. GO term enrichment of biological process from the highest and the lowest salinities (45 versus 8) of time 5 (tc5 and t5) of sampling.

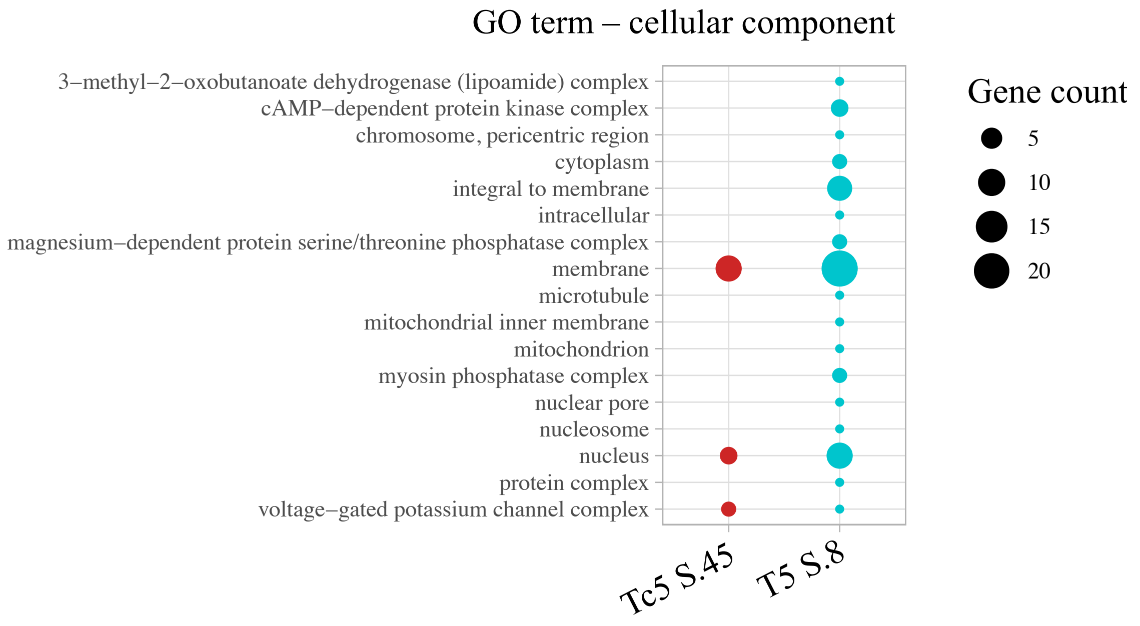

Supplementary Fig. 12. GO term enrichment of cellular component from the highest and the lowest salinities (45 versus 8) of time 5 (tc5 and t5) of sampling.

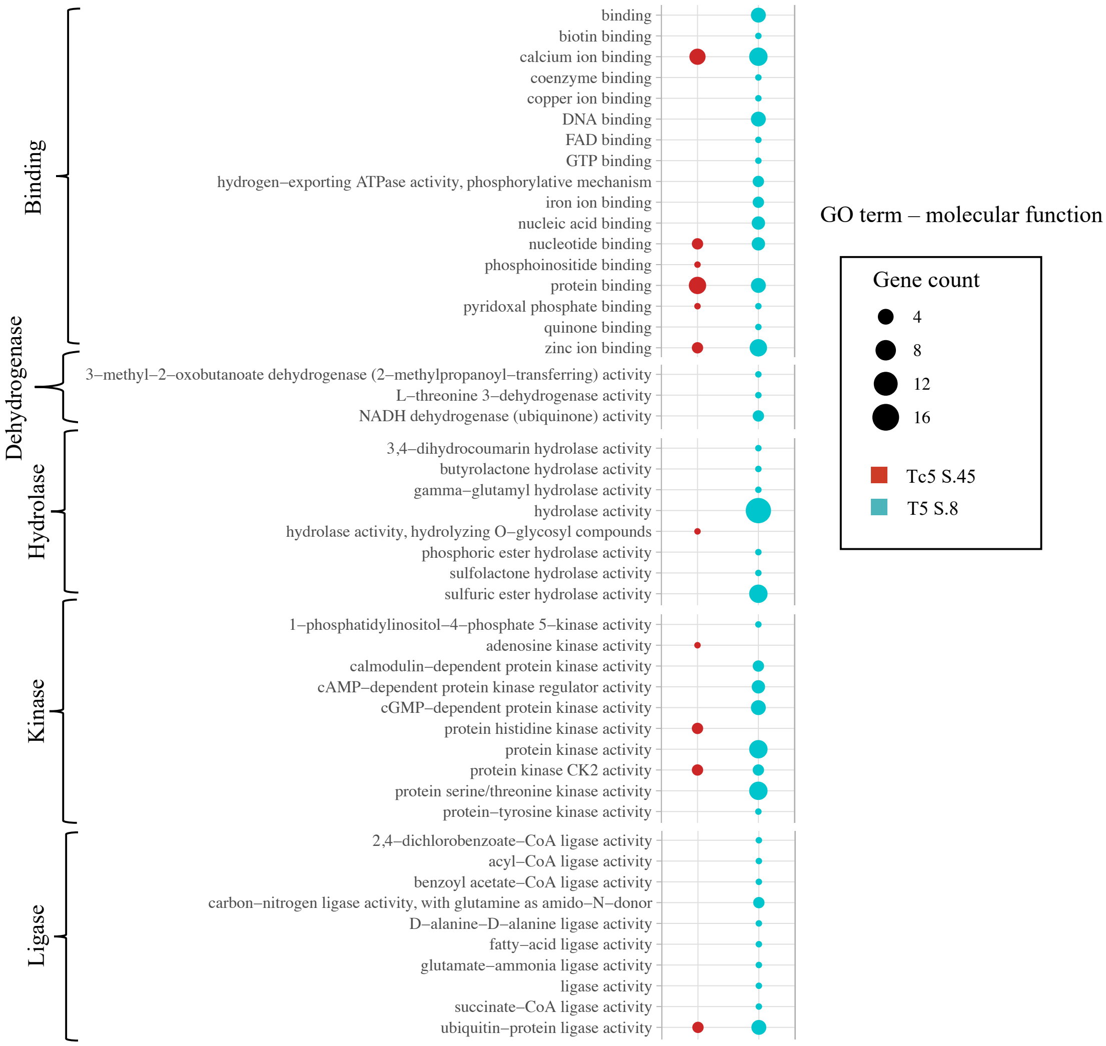

Supplementary Fig. 13. GO term enrichment of molecular function from the highest and the lowest salinities (45 versus 8) of time 5 (tc5 and t5) of sampling. Figure shows the number of genes found in either high or low salinity categorized as binding enzymes, dehydrogenases, hydrolases, kinases and ligases.

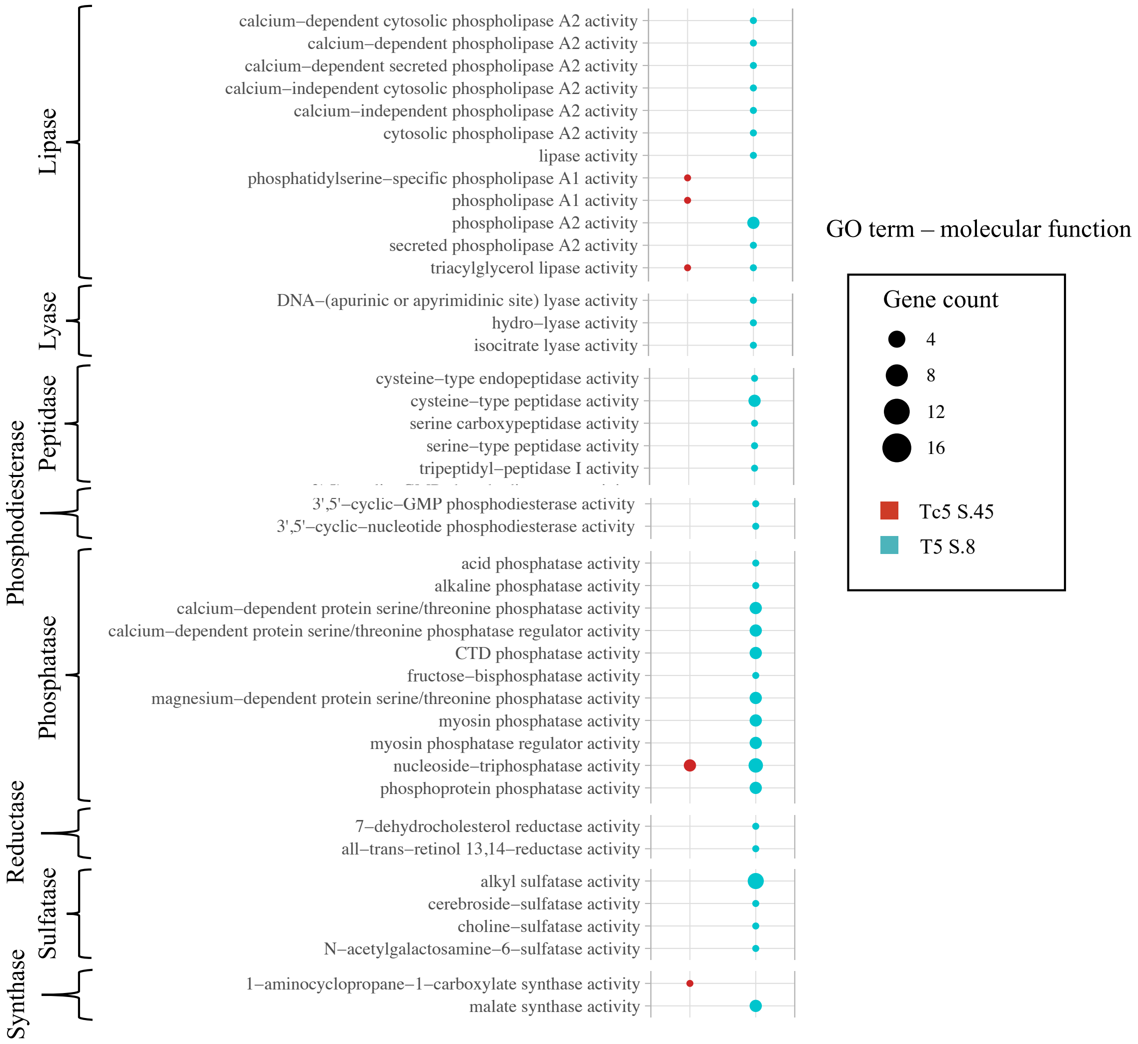

Supplementary Fig. 14. GO term enrichment of molecular function from the highest and the lowest salinities (45 versus 8) of time 5 (tc5 and t5) of sampling. Figure shows the number of genes found in either high or low salinity categorized as lipases, lyases, peptidases, phosphodiesterases, phosphatases, reductases, sulfatases and synthases.

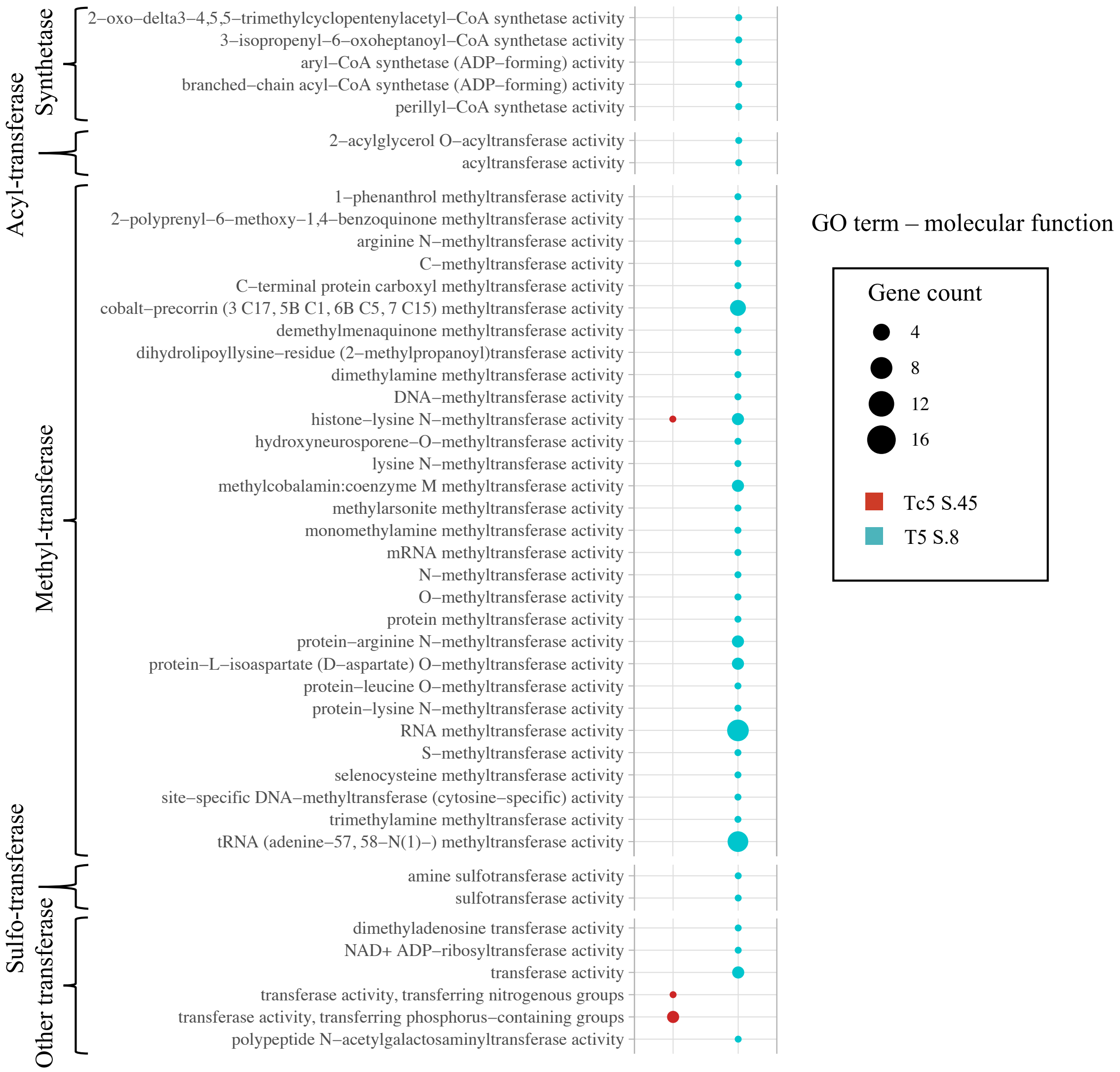

Supplementary Fig. 15. GO term enrichment of molecular function from the highest and the lowest salinities (45 versus 8) of time 5 (tc5 and t5) of sampling. Figure shows the number of genes found in either high or low salinity categorized as synthetases, acyl-, methyl- and sulfo-transferases and other transferases.

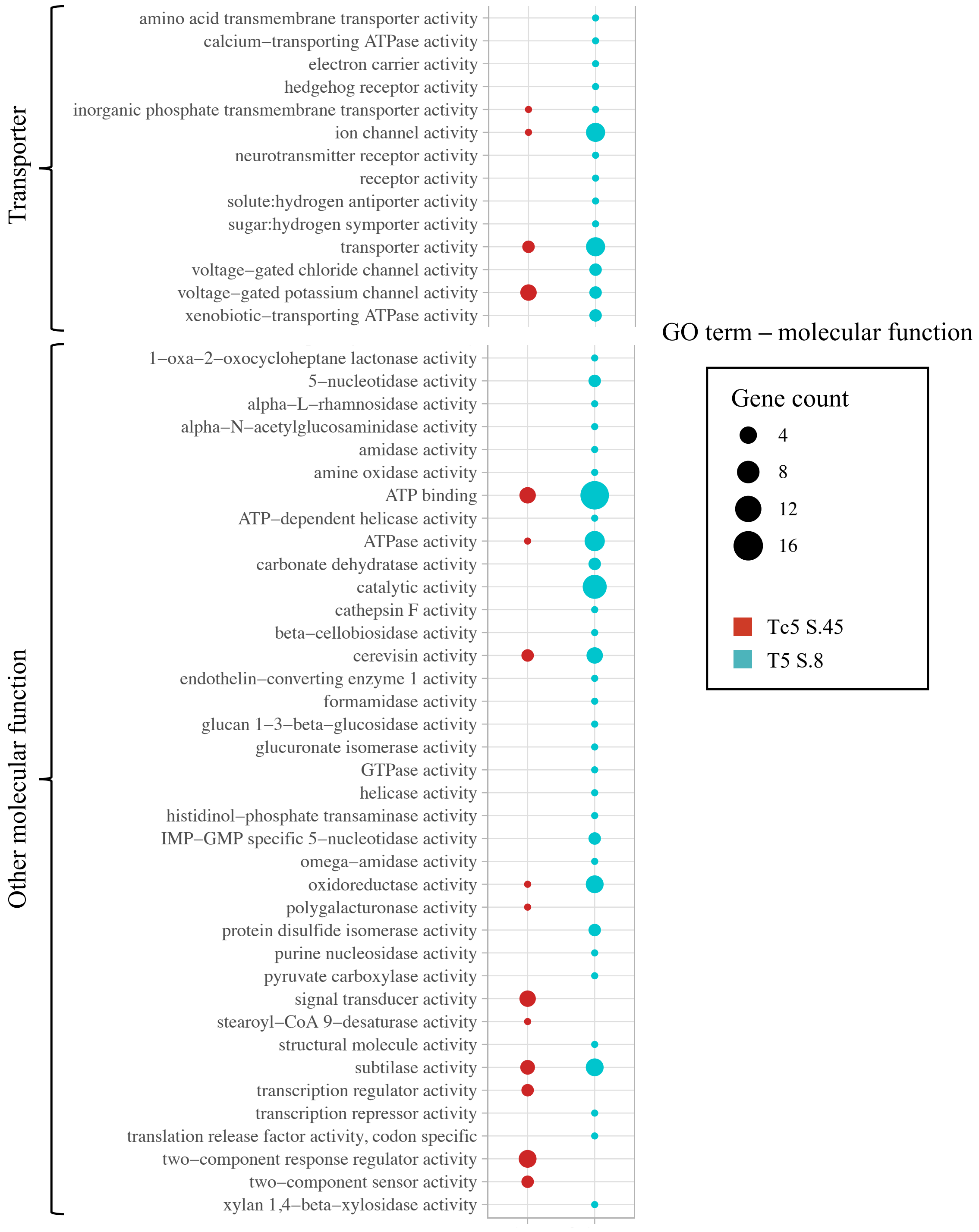

Supplementary Fig. 16. GO term enrichment of molecular function from the highest and the lowest salinities (45 versus 8) of time 5 (tc5 and t5) of sampling. Figure shows the number of genes found in either high or low salinity categorized as transporter enzymes and other enzymes uncategorized.

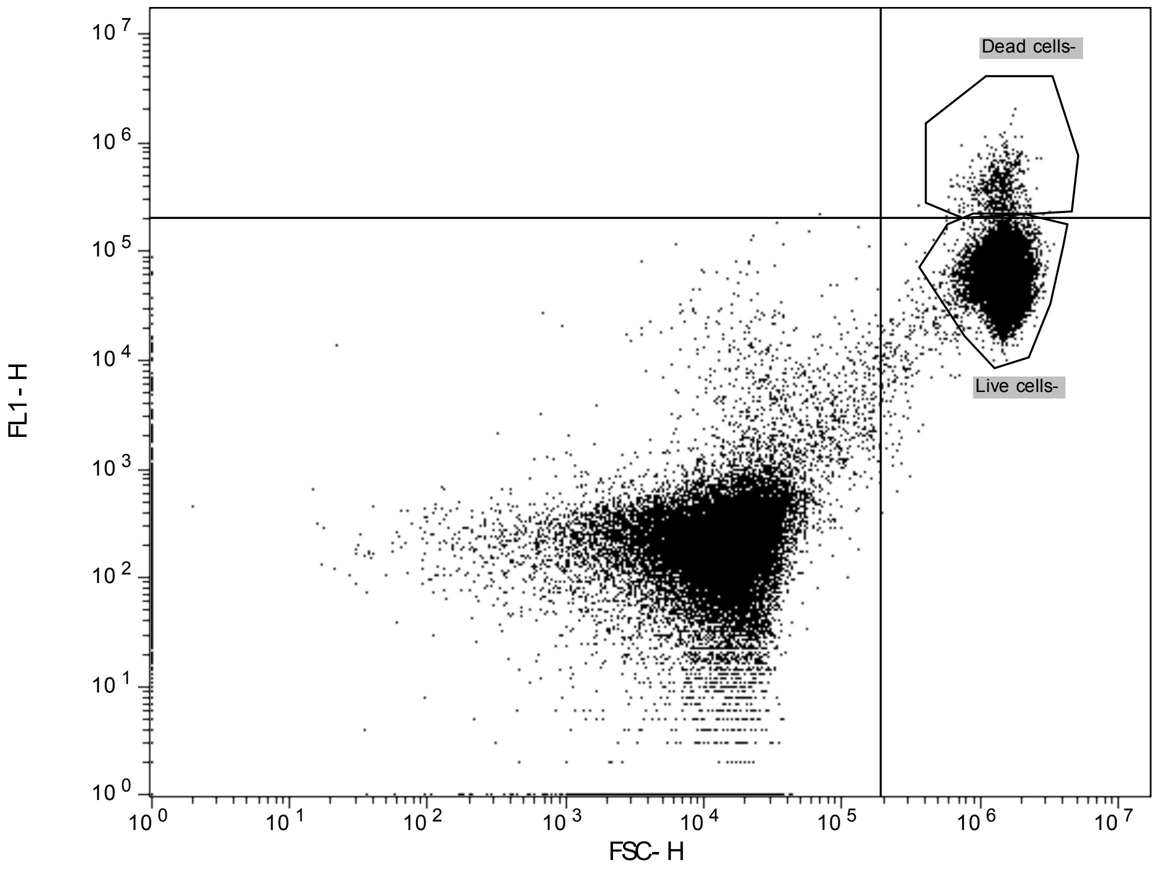

Sample Desc: B5
Gate: -

Label	Events	Percent gated
all	256955	100.00
FSC-H- FL1-H-	182069	70.86
FSC-H+ FL1-H-	71997	28.02
FSC-H- FL1-H+	1	0.00
FSC-H+ FL1-H+	2888	1.12
Live cells-	70946	27.61
Dead cells-	2771	1.08

Supplementary Fig. 17. Example of gates used to enumerate live and dead cells using Flow Cytometry. Dot plot display the number of live and dead cells separated by forward scatter (FSC-H) vs fluorescence intensity (FL1-H).
