# Supplementary material for: Salinity tolerance mechanisms of an Arctic Pelagophyte using comparative transcriptomic and gene expression analysis
Source: Commun Biol. 2022 May 25;5:500. doi: 10.1038/s42003-022-03461-2 (PMC9133084; doi:10.1038/s42003-022-03461-2)
Supplement: Supplementary file 2 — Reporting Summary [file 42003_2022_3461_MOESM2_ESM.pdf]

nature portfolio

Corresponding author(s): Nastasia J. Freyria and Connie Lovejoy

Last updated by author(s): Mar 28, 2022

Reporting Summary

Nature Portfolio wishes to improve the reproducibility of the work that we publish. This form provides structure for consistency and transparency in reporting. For further information on Nature Portfolio policies, see our Editorial Policies and the Editorial Policy Checklist.

Statistics

For all statistical analyses, confirm that the following items are present in the figure legend, table legend, main text, or Methods section.

n/a	Confirmed	
☐	☒	The exact sample size (*n*) for each experimental group/condition, given as a discrete number and unit of measurement
☐	☒	A statement on whether measurements were taken from distinct samples or whether the same sample was measured repeatedly
☐	☒	The statistical test(s) used AND whether they are one- or two-sided<br>*Only common tests should be described solely by name; describe more complex techniques in the Methods section.*
☐	☒	A description of all covariates tested
☒	☐	A description of any assumptions or corrections, such as tests of normality and adjustment for multiple comparisons
☐	☒	A full description of the statistical parameters including central tendency (e.g. means) or other basic estimates (e.g. regression coefficient) AND variation (e.g. standard deviation) or associated estimates of uncertainty (e.g. confidence intervals)
☐	☒	For null hypothesis testing, the test statistic (e.g. *F*, *t*, *r*) with confidence intervals, effect sizes, degrees of freedom and *P* value noted<br>*Give P values as exact values whenever suitable.*
☒	☐	For Bayesian analysis, information on the choice of priors and Markov chain Monte Carlo settings
☐	☒	For hierarchical and complex designs, identification of the appropriate level for tests and full reporting of outcomes
☐	☒	Estimates of effect sizes (e.g. Cohen's *d*, Pearson's *r*), indicating how they were calculated

Our web collection on statistics for biologists contains articles on many of the points above.

Software and code

Policy information about availability of computer code

Data collection	All transcriptome samples are in the DOE JGI Genome Portal under Sequencing Project ID 1253386 and Analysis Project ID 123385, from the Sequence Read Archive (SRP284677-SRP284681). CCMP 2097 reference genome and the annotated genome are available at JGI Genome Portal and PhycoCosm Portal under JGI Project ID 1020062.
Data analysis	All R computer code are available at https://osf.io/h73f8/.

For manuscripts utilizing custom algorithms or software that are central to the research but not yet described in published literature, software must be made available to editors and reviewers. We strongly encourage code deposition in a community repository (e.g. GitHub). See the Nature Portfolio guidelines for submitting code & software for further information.

Data

Policy information about availability of data

All manuscripts must include a data availability statement. This statement should provide the following information, where applicable:

- Accession codes, unique identifiers, or web links for publicly available datasets
- A description of any restrictions on data availability
- For clinical datasets or third party data, please ensure that the statement adheres to our policy

All transcriptome samples are in the DOE JGI Genome Portal under Sequencing Project ID 1253386 and Analysis Project ID 123385, from the Sequence Read Archive (SRP284677-SRP284681). CCMP 2097 reference genome and the annotated genome are available at JGI Genome Portal and PhycoCosm Portal under JGI Project ID 1020062. Computer codes and scripts are available at https://osf.io/h73f8/.

Field-specific reporting

Please select the one below that is the best fit for your research. If you are not sure, read the appropriate sections before making your selection.

☐ Life sciences ☐ Behavioural & social sciences ☒ Ecological, evolutionary & environmental sciences

For a reference copy of the document with all sections, see nature.com/documents/nr-reporting-summary-flat.pdf

Ecological, evolutionary & environmental sciences study design

All studies must disclose on these points even when the disclosure is negative.

Study description	The study was carried out to determine the range and response of an Arctic pelagophyte (CCMP 2097) to changes in salinity, to gain a deeper understanding of the potential genetic capacity of an ice-associated alga to adjust to a range of realistic salinities that would be encountered in its ice influenced habitat. Salinity responses were tested by way of the analysis of 28 transcriptomes to identify key genes that encode enzymes and proteins involved in salinity adaptation. For this, we grew the isolate at salinities from 45 to 8 and using RNA-Seq transcriptomics to describe differential expression profiles under the different salinity conditions.
Research sample	Arctic pelagophyte CCMP2097 was initially isolated from Northern Baffin Bay in June 1998 using a serial selection-dilution technique until a unialgal species was isolated. The isolate was maintained in culture throughout the years in L1 media made up in aged 0.2 µm filtered seawater collected between 300 and 500 m deep from the North Water region, with a salinity of 33-35. Cultures were routinely maintained at ca. 4°C and under a constant illumination.
Sampling strategy	The cultures were subsampled for each salinity condition, with parallel control conditions at the starting salinity of the experiment.
Data collection	We let the cells grow ca. 5 days and harvested the cultures at late exponential phase. For the experimental manipulation, every ca. 5 days, we harvested cells and added increasing amounts of freshwater media until ariving at the final salinity of 8. For a control experiment, we grow cells under the same incubator conditions but maintaied the salinity at 45. we sampled cells at the same time as the salinity treatments and added new medium without changing the salinity. We harvested cells directly from the individual flasks after gentle mixing using a sterile 60 mL syringes (BD, Canada). The sub-samples of the culture were then immediately filtered through a 0.22 µm pore size Sterivex™ Unit (Millipore™ Canada Ltd.). The Sterivex™ filters were then flash frozen in liquid nitrogen and stored at -80*C. Nastasia J. Freyria was responsible and performed the experiment, collected and prepared the samples.
Timing and spatial scale	Both control and salinity change experiment were carried out over 40 days. Cells were harvested the same day of each dilution.
Data exclusions	No data was excluded.
Reproducibility	For a control experiment, we grow cells in the same incubator with identical temperature and light conditions given in the text. The control treatment yielded a total of 14 replicates for the rRNAseq control transcriptomes, the reproducibility was verified with the control cultures all having similar results and clustering togethe. All experimental cultures with salinity c were grown under the same salinity and light condition. The biological replicates results were in close agreement. The aim of this control at the same salinity, was to monitor any time of dilution effects on the cultures.
Randomization	We randomly harvested cells in no particular order among the triplicate. Flasks were placed randomly in the incubator and their position changed over the experiment
Blinding	All scripts were neutral to the design and that there were no anticipated results.

Did the study involve field work? ☐ Yes ☒ No

Reporting for specific materials, systems and methods

We require information from authors about some types of materials, experimental systems and methods used in many studies. Here, indicate whether each material, system or method listed is relevant to your study. If you are not sure if a list item applies to your research, read the appropriate section before selecting a response.

Materials & experimental systems

n/a	Involved in the study	
☒	☐	Antibodies
☒	☐	Eukaryotic cell lines
☒	☐	Palaeontology and archaeology
☒	☐	Animals and other organisms
☒	☐	Human research participants
☒	☐	Clinical data
☒	☐	Dual use research of concern

Methods

n/a	Involved in the study	
☒	☐	ChIP-seq
☐	☒	Flow cytometry
☒	☐	MRI-based neuroimaging

Flow Cytometry

Plots

Confirm that:

- [ ] The axis labels state the marker and fluorochrome used (e.g. CD4-FITC).
- [x] The axis scales are clearly visible. Include numbers along axes only for bottom left plot of group (a 'group' is an analysis of identical markers).
- [ ] All plots are contour plots with outliers or pseudocolor plots.
- [x] A numerical value for number of cells or percentage (with statistics) is provided.

Methodology

Sample preparation	At the time of each harvest, we sampled 2 mL of each culture flask to evaluate proportion of live and dead cells. Each sample was stained with Invitrogen SYTOX™ Green Nucleic Acid Stain (Thermo Fisher Scientific™) to differentiate live and dead cells and enumerated using BD Accuri™ C6 Flow Cytometer.
Instrument	Cells were enumerated using BD Accuri™ C6 Flow Cytometer (BD Biosciences) equipped with the CSampler and 14.7 mW 640 nm Diode Red Laser and 20 mW 488 nm Solid State Blue Laser.
Software	Data were processed with BD CSampler Software.
Cell population abundance	Data acquisition was performed at a slow flow rate (46 µL.min-1) with three agitation and wash cycles between each sample and recalibrated at each time of sampling with 2 µm Fluoresbrite™ beads (BD Trucount™) in filtered seawater to normalized cells counts with flow rate.
Gating strategy	Live and dead cells were separated based on its relative size using forward scattered light (FSC) and relative chlorophyll green fluorescence intensity (FL1) at 530 nm.

- [x] Tick this box to confirm that a figure exemplifying the gating strategy is provided in the Supplementary Information.
